# A Screened IMM-GRU Method for Low-Probability-of-Intercept Maneuvering Target Tracking

**DOI:** 10.3390/s26154691

**Published:** 2026-07-23

**Authors:** Jianjun Rui, Jun Chen, Zhongbin Wang, Wenhao Huang, Shuqiang Xia, Jie Wang

**Affiliations:** 1 School of Electronic and Information Engineering, Nanjing University of Information Science and Technology, Nanjing 210044, China; 2 State Key Laboratory of Mobile Network and Mobile Multimedia Technology, Shenzhen 518055, China; 3 Wireless and Computing Research Institute, ZTE Corporation, Shenzhen 518055, China

**Keywords:** maneuvering target tracking, IMM, Bi-GRU, screened fusion, LPI

## Abstract

**Highlights:**

**What are the main findings?**

- A Screened IMM-GRU framework driven by joint networks and models is proposed for low-probability-of-intercept maneuvering target tracking.
- The proposed screening strategy improves filtering and prediction accuracy by suppressing mismatched or kinematically inconsistent network outputs during model transitions.

**What are the implications of the main findings?**

- Within the motion-model and noise ranges examined in this study, the method improves maneuvering-target tracking accuracy while preserving the interpretability of the IMM multi-model structure.
- Under matched tracking-accuracy requirements, the proposed LPI scheduler requires fewer active radar measurements, thereby reducing radar exposure without relaxing the prescribed accuracy criterion.

**Abstract:**

To address the difficulty of accurate motion-model prespecification and delayed model switching in maneuvering target tracking, this paper proposes a joint model- and network-driven screened Interacting Multiple Model–Gated Recurrent Unit (IMM-GRU) method. In the filtering stage, multiple network outputs are screened using a smooth local reference trajectory constructed from measurements, providing a physically meaningful criterion for rejecting inconsistent estimates while retaining candidates compatible with local track evolution. For radar low-probability-of-intercept (LPI) operation, a difference-enhanced attention Bidirectional GRU (Bi-GRU) classifier identifies target motion modes during radar-silent intervals and supplies model priors for predictor screening. A screened IMM-GRU predictor then selects candidates using model probability, prediction uncertainty, inter-network disagreement, and local kinematic consistency. Cumulative prediction variance and measurement consistency are used to schedule radar radiation. Simulation results show that the proposed filtering and prediction modules improve tracking and extrapolation accuracy. Under the equal-accuracy LPI criterion, the proposed framework achieves the lowest four-scenario average radiation ratio at every prescribed MRMSE level from 40 to 250 m and is the only method that satisfies the 30 m MRMSE requirement in all four scenarios. These results demonstrate improved maneuvering-target tracking within the examined model and noise ranges while reducing unnecessary radar exposure.

## 1. Introduction

The difficulty of maneuvering target tracking lies not only in measurement noise but also in abrupt temporal changes of the target motion mode. Conventional Extended Kalman Filter (EKF), Unscented Kalman Filter (UKF), and Interacting Multiple Model (IMM) filters usually require the Constant Velocity (CV), Constant Acceleration (CA), and Coordinated Turn (CT) motion models and their noise parameters to be specified in advance. When the actual maneuver intensity, turn rate, or model-switching instant is inconsistent with the assumed conditions, the filter often suffers from delayed model transition and accumulated estimation bias [1,2]. In contrast, data-driven tracking methods can learn the local geometric patterns of trajectories directly from measurement sequences without relying entirely on explicit motion equations, and therefore exhibit stronger adaptability in highly maneuvering, nonlinear, and model-incomplete scenarios [3,4]. Recent studies on Transformer, Temporal Convolutional Network-Long Short-Term Memory (TCN-LSTM), and multi-feature-fusion-based maneuvering target tracking also demonstrate that deep temporal models can partly alleviate model mismatch in traditional filters [5,6,7,8].

Among temporal networks, Gated Recurrent Unit (GRU) selectively retains and forgets historical information through update and reset gates. Compared with standard Recurrent Neural Networks (RNNs), GRU is more suitable for long-window measurement sequences; compared with LSTM, it has a simpler structure and fewer parameters, making it convenient for training on large numbers of sliding-window samples and for online deployment [9]. On this basis, Bi-GRU can simultaneously exploit forward motion evolution and backward contextual constraints within a finite historical window. It does not introduce future information outside the window, while enhancing the extraction of velocity changes, curvature variations, and maneuver-transition features inside the window [10]. Therefore, this paper adopts GRU and its bidirectional form, Bi-GRU, as the basic tracking-network units and constructs three types of networks for classification, filtering, and prediction.

However, purely data-driven methods cannot solve all problems in maneuvering target tracking. Although a single end-to-end network can learn complex mappings, it is prone to insufficient interpretability and poor stability under mixed motion modes, out-of-sample maneuvers, or low-Signal-to-Noise-Ratio (SNR) measurements. Recent studies on KalmanNet, Rauch–Tung–Striebel Network (RTSNet), Data-driven Nonlinear State Estimation (DANSE), and Adaptive KalmanNet show that combining state-space models, filtering structures, or Bayesian estimation concepts with neural networks can retain model constraints while improving adaptability to unknown dynamics and noise variations [11,12,13,14,15]. Motivated by these studies, this paper introduces the interacting-multiplemodel idea. Instead of forcing one network to explain all motion patterns, dedicated Bi-GRU networks are constructed for CV, CA, CT, and maneuver switching, and the motion-mode probabilities output by the classification network are used to organize cooperative multi-network estimation.

Furthermore, direct IMM-like probability fusion has an easily overlooked limitation: as long as the output bias of a mismatched network is sufficiently large, even a low probability may drag the final estimate through weighted averaging. In the prediction phase, such bias also enlarges uncertainty through inter-network disagreement terms in the mixture variance, causing the subsequent scheduler to enter the radiation state prematurely. To address this issue, this paper introduces a screening mechanism into the IMM-GRU framework. The screened filtering algorithm is based on the convex feasible domain formed by the outputs of four filtering networks and uses the Continuous-Acceleration Rauch-Tung-Striebel (CA-RTS) smoother output as a reference to suppress estimates that deviate from the consensus of the network family. The prediction algorithm selects more reliable prediction networks for fusion by jointly considering classification probability, prediction variance, inter-network disagreement, and local kinematic consistency. This design preserves the interpretability of the IMM multi-model structure while avoiding interference from low-confidence networks in direct fusion [6,16,17].

In addition to tracking accuracy, active radar must also account for low-probability-of-intercept performance; more generally, sensing-system design requires sensing performance to be assessed jointly with resource allocation and application requirements [18]. This paper reduces the interception risk of radar signals in maneuvering target tracking by controlling the radar radiation timing. Continuous radar radiation provides continuous measurements, but also increases exposure probability. Reducing the number of active radiations is beneficial for LPI performance, but makes the maneuvering segment more dependent on model prediction; if the model-transition response is not timely, track error may accumulate rapidly or even diverge [19,20,21,22]. Therefore, this paper combines the Screened IMM-GRU predictive tracking network with the filtered tracking network: when the radar does not radiate, the prediction network maintains the track and accumulates prediction risk; once the risk increases, radiation is restored to acquire real measurements, and the screened filtering network then performs rapid correction.

Based on the above considerations, the main contributions of this paper are summarized as follows.

- A difference-enhanced attention Bi-GRU classification network is developed for motion-mode recognition of maneuvering targets. By jointly exploiting position, first-order differences, second-order differences, and attention pooling, the network identifies CV, CA, CT, and maneuver-transition modes and provides reliable mode priors for subsequent IMM-GRU fusion.
- A Screened IMM-GRU filtering algorithm is proposed to improve tracking accuracy under model switching and strong maneuvers. The method uses a CA-RTS local smoothing reference and projects it onto the convex feasible domain formed by multiple GRU tracking outputs, thereby suppressing the influence of low-confidence mismatched networks in direct probability fusion.
- A Screened IMM-GRU prediction-filtering LPI tracking framework is constructed for radar-silent maneuvering target tracking. During non-radiation intervals, reliable prediction networks are selected according to mode probability, prediction uncertainty, and local kinematic consistency; once the prediction risk increases, radar radiation is restored and screened filtering is used for rapid correction.

## 2. Three Types of Bi-GRU Network Models for Maneuvering Target Tracking

**Figure 1:**
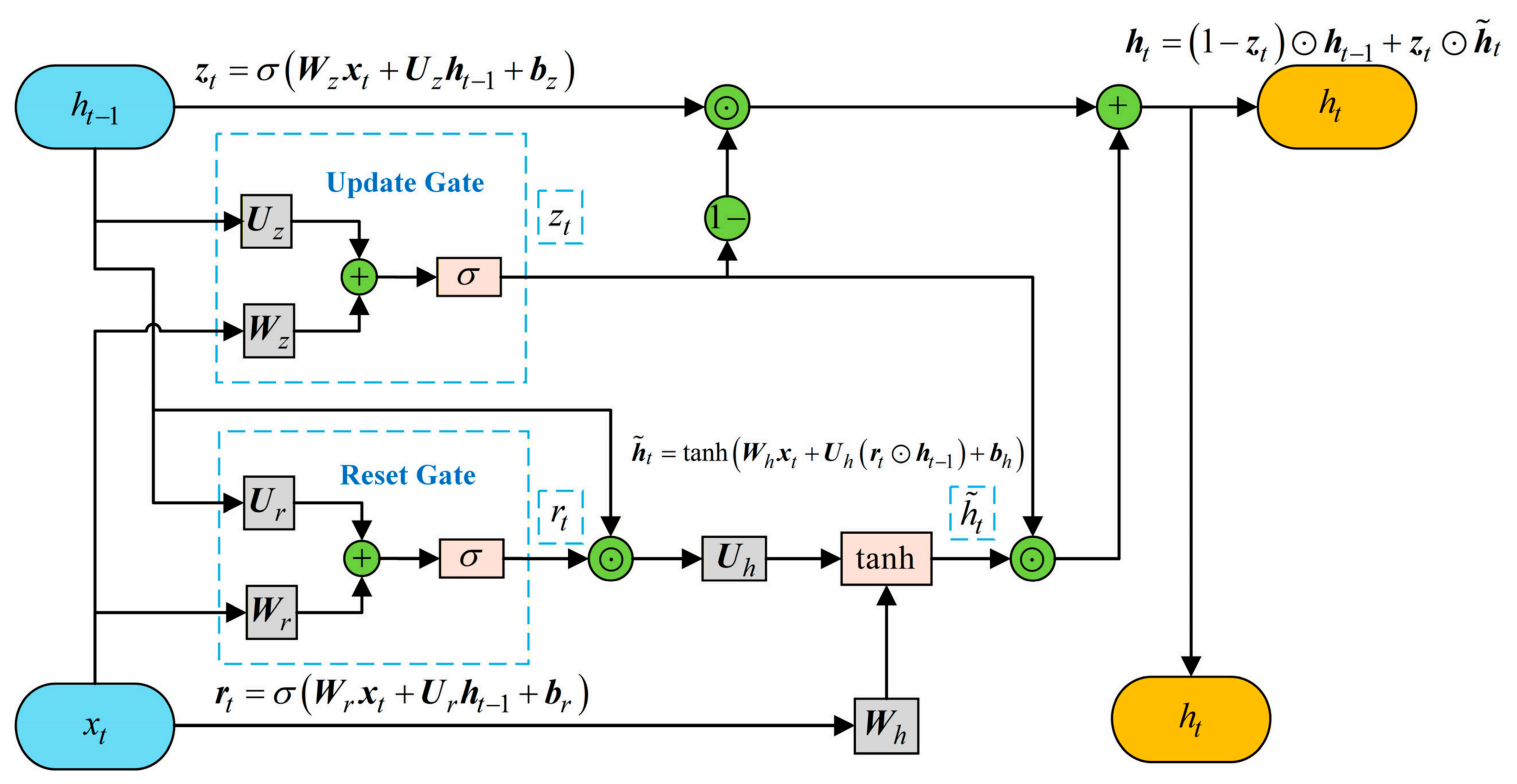
Structure of the GRU unit.

In maneuvering target tracking, a tracker must maintain existing velocity or turning trends during stable segments and respond rapidly to motion-mode changes during maneuvering segments. As shown in Figure 1, a GRU unit is mainly composed of an update gate, a reset gate, and a candidate hidden state. Through the gating mechanism, it adaptively regulates the retention, forgetting, and updating of historical motion information, thereby continuously modeling the target motion trend. In particular, when the update gate is small, the network tends to maintain the existing motion trend; when the update gate is large, it becomes more sensitive to current measurement changes. This behavior is consistent with the basic requirements of maneuvering target tracking.

Building upon the GRU unit, Bi-GRU jointly models the measurement sequence within a sliding window through two distinct GRU branches—a forward branch and a backward branch. The forward branch captures the temporal evolution of target motion, while the backward branch provides complementary contextual constraints within the window, thereby enhancing the capability to extract features related to velocity changes, curvature variations, and model switching [10]. Compared to a unidirectional GRU, Bi-GRU is better suited for performing motion pattern recognition, trajectory smoothing, and short-term prediction within a limited historical window. Consequently, this paper adopts Bi-GRU as the core architectural framework to construct separate classification, filtering, and prediction networks, thereby laying the foundation for a subsequent LPI tracking algorithm driven jointly by networks and models.

### 2.1. Data Preprocessing

Maneuvering-target tracking is inherently three-dimensional. In this study, the original three-dimensional problem is projected onto the horizontal range–azimuth plane so that the target’s turning, acceleration, and motion-mode transitions can be analyzed more directly and visualized more clearly. For a range–azimuth–elevation measurement, the slant range is first converted into horizontal range, after which Equation (2) maps the horizontal range and azimuth into the x-y coordinates used by the tracker.

Let the target measurement acquired by radar radiation at time k be defined as $z_{k}={\left[d_{k},\theta_{k}\right]}^{T}$, where $d_{k}$ and $\theta_{k}$ denote the range and bearing angle of the target relative to the coordinate origin, respectively. The measurement equation is expressed as:

(1) $$z_{k}=Hx_{k}+v$$

where H denotes the measurement matrix; $v={\left[v_{d},v_{\theta }\right]}^{T}$ denotes the measurement noise, which is zero-mean Gaussian white noise; and $v_{d}$ and $v_{\theta }$ denote range noise and bearing-angle noise, with standard deviations $\sigma_{d}$ and $\sigma_{\theta }$, respectively.

Before the measurement enters the network, the polar measurement is first converted into a Cartesian measurement:

(2) $$z_{k}^{c}={\left[z_{xk},z_{yk}\right]}^{T}={\left[d_{k}\cos\theta_{k},d_{k}\sin\theta_{k}\right]}^{T}$$

Then, min-max normalization is performed:

(3) $${\hat{\bar{z}}}_{j}=\frac{z_{j}^{c}-z_{min}}{z_{max}-z_{min}},\;\;\;z_{min}=\underset{j}{min}z_{j}^{c},\;\;\;z_{max}=\underset{j}{max}z_{j}^{c}$$

where the normalized coordinate is evaluated for *j* = *k* − *L* + 1, …, *k*, and the minimum and maximum are computed over the current sliding window. This affine mapping reduces scale variation across trajectories and provides a common input range for all networks; the network output is subsequently transformed back to physical coordinates using the same window statistics. Equations (1)–(3) specify the measurement representation, coordinate conversion, and normalization used in this study, following the state-estimation conventions in [2,23].

### 2.2. Principles of Classification Network

In general, the basic target motion models mainly include the constant-velocity model, the coordinated-turn model, and the constant-acceleration model. In this paper, a motion model constructed by selecting the switching process between two different models from the three basic motion models is referred to as the maneuver model. If the switching instant is $k_{s}$ and the models before and after switching are $m_{1}$ and $m_{2}$, respectively, it can be expressed as:

(4) $$m_{k}=\left\{\begin{array}{cc} m_{1}, & 0\leq k<k_{s},\\ m_{2}, & k_{s}\leq k\leq N-1, \end{array}\right.\;\;\;m_{1},m_{2}\in \{\mathrm{CV},\mathrm{CA},\mathrm{CT}\},\;\;\;m_{1}\neq m_{2}\;$$

The classification network is used to determine whether the current measurement window is closer to CV, CA, CT, or the maneuver model. Its output mode probabilities also serve as important priors for the subsequent screened fusion of the filtering and prediction networks. To enable the network to explicitly identify maneuver transitions, for the input normalized two-dimensional measurement ${\hat{\bar{z}}}_{j}$, the classification network performs difference enhancement before the data enter the network layer, constructing the first- and second-order differences:

(5) $$\Delta {\hat{\bar{z}}}_{j}={\bar{z}}_{j}-{\bar{z}}_{j-1},\;\;\;\;\;\;\Delta^{2}{\hat{\bar{z}}}_{j}=\Delta {\hat{\bar{z}}}_{j}-\Delta {\hat{\bar{z}}}_{j-1}$$

Difference enhancement has a direct kinematic interpretation: position describes the local trajectory geometry, the first-order difference approximates velocity variation, and the second-order difference captures acceleration or curvature variation. Equation (5) therefore exposes motion cues that are not available from the position sequence alone and improves discrimination among CV, CA, CT, and transition windows.

For three-dimensional motion-mode recognition, the modification is confined to the feature interface rather than the temporal architecture. For a Cartesian position vector with x-, y-, and z-components, the position, first-order-difference, and second-order-difference vectors are concatenated, increasing the classifier input from six to nine channels. The three-layer Bi-GRU, attention-pooling module, and four-class softmax output are retained. The CV class includes constant three-axis velocity, including steady climb or descent; the CA class includes three-axis acceleration; the CT class is generalized to coordinated rotation about an arbitrary spatial turn axis; and windows containing transitions among these spatial modes, including coupled turn-climb changes, are assigned to the maneuver class. For parameter transfer, the recurrent hidden-to-hidden weights and attention parameters learned from planar data are retained; the x- and y-related columns of the first-layer input-to-hidden matrices are copied, the new z-related columns are initialized, and the complete classifier is then jointly fine-tuned on spatial trajectories. This procedure preserves the temporal features learned in the horizontal plane while enabling recognition of genuinely three-dimensional mode transitions.

**Figure 2:**
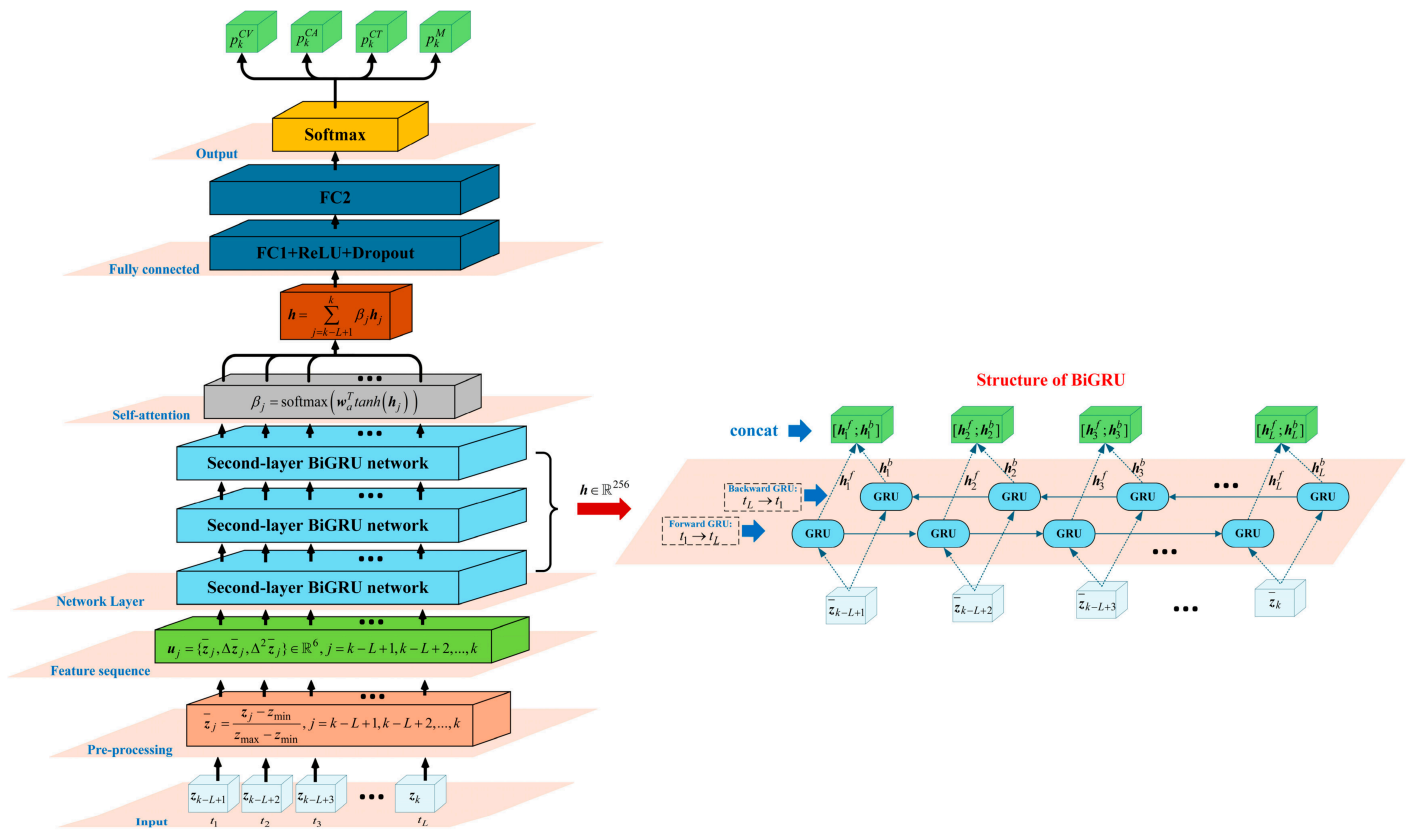
Structure of the classification network.

As shown in Figure 2, the classification network employs a three-layer bidirectional GRU with a hidden dimension of 256. To enable the network to focus on the segments that best characterize the motion mode, such as local curvature changes when turning begins near the end of a stable segment [5], an attention-pooling mechanism is adopted at the output of the classification network. The process of attention pooling is shown below.

1. First, the activation function tan(⋅) is used to calculate each hidden state output by the network layer, obtaining the score of each hidden state:
  (6) $$e_{j}=w_{a}^{T}\tanh(h_{j})$$
2. Then, the softmax function is used to calculate each attention weight:
  (7) $$\beta_{j}=\frac{\exp(e_{j})}{\sum_{q=k-L+1}^{k}\exp(e_{q})}$$
3. Finally, the attention weights and hidden states are weighted and summed to obtain the output of the self-attention mechanism:
  (8) $$h=\sum_{j=k-L+1}^{k}\beta_{j}h_{j}$$

The attention output is passed through two fully connected layers, and a final softmax layer produces the four class probabilities. Equations (6)–(9) follow the conventional additive-attention construction on Bi-GRU hidden states [5,9,10]: scalar compatibility scores are normalized, the hidden states are pooled with the resulting weights, and the pooled representation is mapped to class probabilities.

(9) $$p_{k}={\left[p_{k}^{CV},p_{k}^{CA},p_{k}^{CT},p_{k}^{M}\right]}^{T},\;\;\;\;\;\;\sum_{i}p_{k}^{i}=1$$

In addition, for training the classification network, the motion-mode label sequence k−L+1,…,k within the sliding window ($Y_{k}=\{y_{k-L+1},\ldots ,y_{k}\}$) needs to be defined. The motion-mode labels are determined by the true model indices within the window. Let $m_{j}\in \{0,1,2\}$ denote constant velocity, constant acceleration, and coordinated turn, respectively. The window label $y_{k}$ is defined as:

(10) $$y_{k}=\left\{\begin{array}{cc} m_{j}, & y_{k-L+1}=\cdots =y_{k},\\ 3, & \mathrm{Maneuver}. \end{array}\right.$$

During classification-network training, the loss is characterized by cross entropy:

(11) $$L_{c}=-\frac{1}{B}\sum_{i=1}^{B}\sum_{m=0}^{3}I(y_{i}=m)\log p_{i}^{m}$$

where $y_{i}$ is the true label of the *i*-th sample and $I(y_{i}=m)$ is an indicator that equals 1 when $y_{i}$ matches class *m* and 0 otherwise. Equation (10) defines the window-level labeling rule used in this work, whereas Equation (11) is the standard multiclass cross-entropy loss [9,10].

### 2.3. Principles of Filtering Network

The Bi-GRU filtering network is used to recover the target-position trajectory from the most recent noisy Cartesian measurement window when the current radar measurement has arrived, especially to estimate the current position at the end of the window. Therefore, the network can be regarded as a data-driven local sequence smoother and current-state estimator.

**Figure 3:**
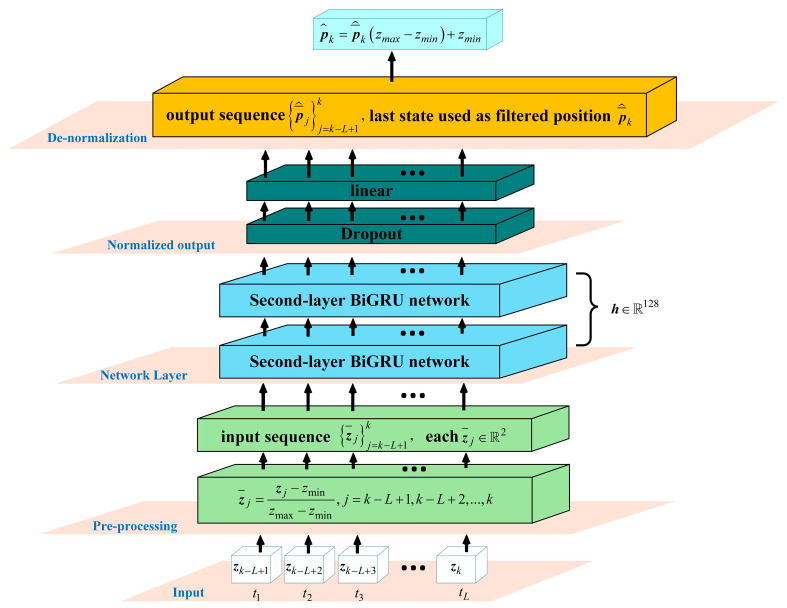
Structure of the filtering network.

Based on the above design goals, this paper constructs the filtering network structure shown in Figure 3. The normalized two-dimensional measurement sequence ${\hat{\bar{Z}}}_{k}\in {\mathbb{R}}^{L\times 2}$ is first input into a two-layer bidirectional GRU network, with each layer having a hidden dimension of 128. Then, it passes through a Dropout layer and a linear mapping layer to obtain the normalized position estimation sequence.

In the final output stage, only the last element of ${\hat{\bar{P}}}_{k}\in {\mathbb{R}}^{L\times 2}$ is retained as the position estimate for the current sample and transformed back to physical coordinates. Equation (12) is the inverse of the min-max normalization in Equation (3), evaluated at the window endpoint.

(12) $${\hat{p}}_{k}={\hat{\bar{p}}}_{k}(z_{\max}-z_{\min})+z_{\min}$$

As shown in Figure 3, the filtering network adopts a sequence-to-sequence output format, but in application, the output results at the end of the window are mainly used. This design allows the network to learn the overall continuity of the trajectory within the window and enhances the accuracy of the position estimation at the current time. Therefore, this paper further employs endpoint-weighted regression loss to train the filtering network.

For training the filtering networks, a corresponding filtering network is trained separately for each motion model. For example, CV, CA, and CT use single-model trajectory windows for training, whereas the maneuver model uses trajectory windows in which model switching occurs. Let the batch size be B, then the training loss function is:

(13) $$L_{f}=\frac{1}{B}\sum_{i=1}^{B}{\left[\frac{1-\alpha }{L}\sum_{j=1}^{L-1}{\left\|{\hat{p}}_{i,j}-p_{i,j}\right\|}_{2}^{2}+\alpha {\left\|{\hat{p}}_{i,L}-p_{i,L}\right\|}_{2}^{2}\right]}^{1/2}$$

where *α* = 0.6. This value was selected on the independent validation set to prioritize the endpoint error used for online tracking while retaining 40% sequence supervision to regularize the trajectory shape. It is fixed for all four filtering networks.

For three-dimensional filtering, the input and output sequences are replaced by their L-by-3 Cartesian counterparts. Only the input interface of the first Bi-GRU layer and the final linear output layer require dimensional expansion; the two-layer recurrent structure, hidden dimension, Dropout operation, and endpoint-weighted objective remain unchanged, with the regression norm evaluated jointly over x, y, and z. The recurrent hidden-to-hidden matrices are transferred directly from the planar networks, the x- and y-related columns and rows of the input and output mappings are copied, and only the new z-related parameters are initialized before joint fine-tuning. The four model-specific filters are then trained with spatial CV, CA, and CT trajectories, together with climb/descent and coupled transition windows, so that vertical motion is learned jointly with horizontal dynamics rather than processed as an independent scalar channel.

### 2.4. Principles of Prediction Network

The prediction network provides reliable prediction outputs for the radar silent phase in LPI tracking. In a related passive-sensing setting, accumulated Doppler and angle observations have been used to reconstruct target trajectories and predict short-term blockage events [24]. After the radar stops active radiation, the system cannot obtain current real measurements and can only maintain the track using the historical buffer and its own prediction results. Therefore, the prediction network must output both the next-step position mean and the corresponding uncertainty.

Unlike the filtering network, whose input consists entirely of real measurements, the input sliding window of the prediction network gradually contains prediction points backfilled by the network itself. To explicitly distinguish real measurements from closed-loop prediction results, this paper constructs five-dimensional features for each buffer point, including position, real-measurement flag, variance trace, and prediction age, so that the network can perceive how input reliability changes with silent duration.

(14) $$\xi_{j}={\left[{\hat{\bar{x}}}_{j},{\hat{\bar{y}}}_{j},r_{j},log{\hat{\bar{v}}}_{j},a_{j}\right]}^{T}$$

where ${\hat{\bar{x}}}_{j}$ and ${\hat{\bar{y}}}_{j}$ are the normalized position coordinates, $r_{j}\in \{0,1\}$ indicates whether the point is a real measurement, ${\hat{\bar{v}}}_{j}$ is the variance trace normalized by the squared window scale, and $a_{j}$ is the prediction age. Equation (14) concatenates these five features for closed-loop prediction; the logarithm compresses the dynamic range of the nonnegative variance term.

**Figure 4:**
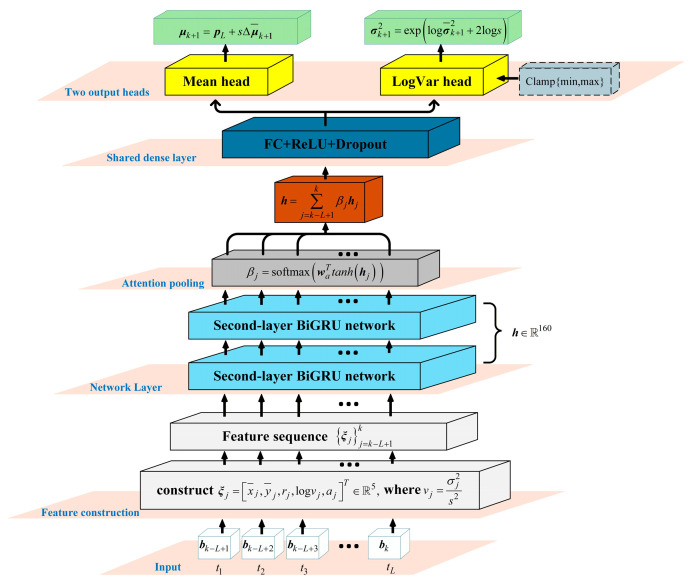
Structure of the prediction network.

Based on the above features, this paper designs the prediction network as a heteroscedastic one-step predictor with a time attention mechanism, and its structure is shown in Figure 4.

As shown in Figure 4, the constructed feature sequence first passes through two layers of a bidirectional GRU network with a hidden dimension of 160 to extract temporal motion information from the historical window. Then, temporal attention pooling is used to obtain the hidden state representation most relevant to the next extrapolation step. This representation is then passed through a shared fully connected layer and input to the mean header and log-variance header, respectively. The former outputs the normalized displacement mean $\Delta {\hat{\bar{\mu }}}_{k+1}$, and the latter outputs the normalized log-variance $\log{\hat{\bar{\sigma }}}_{k+1}^{2}$, respectively:

(15) $${\hat{\bar{\mu }}}_{k+1}=W_{\mu }\phi (h)+b_{\mu },\;\;\;\log{\hat{\bar{\sigma }}}_{k+1}^{2}=\mathrm{clip}(W_{\sigma }\phi (h)+b_{\sigma },-8,5)$$

In Equation (15), the log-variance is clipped to [−8, 5], not to the decimal value 8.5. The interval is deliberately broad but finite: it prevents numerical underflow or overflow and unrealistically extreme confidence during recursive prediction without tightly constraining the learned uncertainty.

(16) $$\mu_{k+1}=p_{L}+s\Delta {\hat{\bar{\mu }}}_{k+1},\;\;\;\sigma_{k+1}^{2}=\exp(\log{\hat{\bar{\sigma }}}_{k+1}^{2}+2\log s)$$

Equation (16) follows from affine de-normalization: displacement scales linearly with the window span, whereas variance scales with its square. Prediction-network training proceeds from single-step learning to multi-step recursive rollout. During the first stage, the input buffer contains only real historical measurements, allowing the predictor to learn the local motion relation before it is exposed to its own recursively generated outputs. The single-step objective is the heteroscedastic Gaussian negative log-likelihood:

(17) $$L_{\mathrm{nll}}=\frac{1}{2}\sum_{r\in \{x,y\}}\left[\frac{{\left(p_{k+1,r}-\mu_{k+1,r}\right)}^{2}}{\sigma_{k+1,r}^{2}}+\log\sigma_{k+1,r}^{2}\right]$$

This loss jointly calibrates the predicted mean and variance. An underestimated variance is strongly penalized when the mean error is large, whereas an excessively large variance is penalized by the logarithmic term. Equation (17) is the negative log-likelihood of a diagonal Gaussian after constants independent of optimization are omitted [25].

The second stage performs multi-step rollout training with scheduled sampling [26], so that the predictor gradually encounters the same mixture of measured and self-generated inputs that occurs during radar silence. For a maximum horizon H, the discounted rollout loss is

(18) $$L_{p}=\sum_{h=1}^{H}\gamma^{h-1}L_{\mathrm{nll}}^{\left(h\right)}$$

where the discount factor is *γ* = 0.97. At the 30th step, the weight remains *γ*^29^ ≈ 0.41; this keeps the full 3 s rollout supervised while preventing the less reliable distant steps from dominating the loss. The value is fixed for all prediction networks.

For three-dimensional prediction, each buffer feature is expanded to six components by adding the normalized vertical coordinate, namely three Cartesian coordinates together with the real-measurement flag, logarithmic variance feature, and prediction age. The Bi-GRU and temporal-attention blocks are unchanged, whereas the mean and log-variance heads are expanded from two to three outputs. The heteroscedastic likelihood is summed over the x, y, and z axes, and the scalar variance feature written back to the buffer is obtained from the trace of the three-dimensional position covariance normalized by the squared spatial scale. Multi-step scheduled-sampling training is performed on spatial turns, climb/descent motion, and coupled turn-climb rollouts. The recurrent and attention parameters can be inherited from the planar predictor; the x-y input/output parameters are copied, the z-related parameters are newly initialized, and all layers are jointly fine-tuned so that cross-axis coupling is learned during recursive prediction.

## 3. Screened IMM-GRU Fusion Filtering and Prediction Algorithms

Section 2 presents the three types of Bi-GRU networks for classification, filtering, and prediction. If the outputs of the four networks are directly fused according to classification probabilities, low-confidence networks near model switching may still drag the result with small weights, and inter-network disagreement in the prediction phase may amplify uncertainty. Therefore, this section introduces a screening mechanism while retaining the IMM multi-model parallelism: the filtering algorithm uses the CA-RTS smoother output as a reference and projects it onto the convex feasible domain of the network outputs to obtain the final estimate; the prediction algorithm selects reliable prediction networks using probability, variance, and local kinematic consistency.

### 3.1. Screened IMM-GRU Filtering-Tracking Algorithm

#### 3.1.1. Basic Fusion Model

The traditional IMM handles unknown maneuvering modes by running multiple candidate models in parallel and fusing the results of their subfilters. This paper retains this multi-model parallel idea but replaces the model-matched EKF subfilters with data-driven Bi-GRU networks, reducing dependence on exact motion equations and fixed model parameters [4].

This structure has three advantages. First, Bi-GRU networks can learn measurement-to-state mappings for different motion modes from samples. Second, the classification network can directly estimate mode probabilities using velocity, acceleration, and curvature changes within the window. Third, multi-network outputs and fusion weights provide clearer physical interpretation than a single black-box network.

Let the four filtering network functions be $f_{0},f_{1},f_{2},f_{3}$, corresponding to the CV, CA, CT, and maneuver models, respectively. When the radar actively radiates, the system can obtain a new real measurement at each time *k*. After the latest measurement is written into the sliding window, the four filtering networks perform parallel filtering and produce four candidate position estimates:

(19) $${\hat{p}}_{k}^{\left(i\right)}=f_{i}({\hat{\bar{Z}}}_{k}),\;\;\;i=0,1,2,3.$$

The classification network outputs the mode probability $p_{k}^{\left(i\right)}$ for the same window. The most direct IMM-GRU fusion method is probability weighting:

(20) $${\hat{p}}_{k}=\sum_{i=0}^{3}p_{k}^{\left(i\right)}P_{k}^{\left(i\right)}$$

Although direct probability weighting preserves the basic idea of IMM, low-probability mismatched networks still enter the fusion with small weights. If such a network produces a large bias near model switching, the fused mean may still be dragged. Therefore, credible-output screening is necessary before fusion. Equations (19) and (20) are, respectively, the parallel expert-output definition and the conventional posterior-probability mixture inherited from the IMM principle [2].

#### 3.1.2. Screened Filtering Based on the CA-RTS Smoother

To address the problem that low-probability mismatched networks may drag the estimate, this paper implements screened filtering by using the CA-RTS smoother output as a reference based on the convex feasible domain of network outputs.

Following the candidate definitions in Section 3.1.1, let the output of the i-th filtering network at time k be the corresponding CV, CA, CT, or maneuver estimate. Their convex feasible domain is defined as

(21) $$D_{k}=\left\{p\in {\mathbb{R}}^{2}\left|p=\sum_{i=0}^{3}\lambda_{i}{\hat{P}}_{k}^{\left(i\right)},\;\lambda_{i}\geq 0,\;\sum_{i=0}^{3}\lambda_{i}=1\right.\right\}$$

The feasible domain is the convex hull of the four expert positions: every admissible output is a nonnegative, unit-sum combination of the network estimates. A reference point outside this domain is not supported by the current expert family and is therefore not adopted directly, even when it lies close to an individual noisy measurement.

The CA-RTS smoother consists of forward filtering and backward smoothing. The forward filtering is based on a Kalman filter under the CA model. The target state vector under the CA model can be expressed as

(22) $$s_{k}={\left[x_{k},v_{xk},a_{xk},y_{k},v_{yk},a_{yk}\right]}^{T}$$

Let the sampling interval be *T*. The state transition equation of $s_{k}$ can be written as:

(23) $$s_{k+1}=F_{\mathrm{CA}}s_{k}+w_{k}$$

where the model state-transition matrix is

$$F_{\mathrm{CA}}=\left[\begin{array}{cccccc} 1 & T & T^{2}/2 & 0 & 0 & 0\\ 0 & 1 & T & 0 & 0 & 0\\ 0 & 0 & 1 & 0 & 0 & 0\\ 0 & 0 & 0 & 1 & T & T^{2}/2\\ 0 & 0 & 0 & 0 & 1 & T\\ 0 & 0 & 0 & 0 & 0 & 1 \end{array}\right],$$

the process noise is $w_{k}∼N(0,Q_{s})$, and $q_{s}$ denotes the auxiliary process-noise intensity. Then the discrete covariance of $w_{k}$ can be written as

(24) $$Q_{s}=q_{s}\mathrm{diag}(Q_{0},Q_{0})$$

where $Q_{0}=\left[\begin{array}{ccc} T^{5}/20 & T^{4}/8 & T^{3}/6\\ T^{4}/8 & T^{3}/3 & T^{2}/2\\ T^{3}/6 & T^{2}/2 & T \end{array}\right]$.

Because the Kalman forward filtering and RTS backward smoothing based on the CA model are both performed in the Cartesian coordinate system, the measurement-noise covariance matrix in the polar coordinate system must be converted to Cartesian space through local linearization, namely:

(25) $$R_{k}^{c}=J_{k}R_{k}J_{k}^{T}$$

where $R_{k}$ is the range-bearing measurement-noise covariance, and $J_{k}$ is the Jacobian matrix of the coordinate transformation in Equation (2) at the current measurement. From Equation (1), the measurement equation here can be written as $z_{k}^{c}=H_{s}s_{k}+v_{k}^{c}$, where the measurement matrix $H_{s}$ extracts only the two-dimensional position components from the auxiliary state. The state definition, CA transition, discrete process covariance, and Jacobian covariance propagation in Equations (22)–(25) follow the standard linear-Gaussian state-space formulation [2,23].

In a complete three-dimensional implementation, the CA-RTS auxiliary state is expanded to nine components, with position, velocity, and acceleration along each Cartesian axis. The state-transition and process-noise matrices become block diagonal with three CA blocks. A range-azimuth-elevation measurement is transformed to Cartesian coordinates, its covariance is propagated through the corresponding three-dimensional spherical-to-Cartesian Jacobian, and the measurement matrix extracts the x-, y-, and z-position components. The RTS recursion then produces a three-component local reference at the same fixed lag n*T*.

Based on $F_{\mathrm{CA}}$, $Q_{s}$, $H_{s}$, and $R_{k}^{c}$, standard Kalman filtering recursions can be used to obtain the filtered state $s_{k}^{f}$, filtered covariance $P_{k}^{f}$, and the corresponding one-step predictions $s_{k}^{-}$ and $P_{k}^{-}$.

The RTS backward smoothing is then performed. For each state time *k*, $m_{k}=\min(N,k+n)$ is taken as the farthest available future measurement point, and RTS backward recursion is performed from $m_{k}$ to *k*, where *n* is the positive integer fixed-lag order. The backward gain is

(26) $$G_{j}=P_{j}^{f}F_{s}^{T}{\left(P_{j+1}^{-}\right)}^{-1}$$

The corresponding smoothed state satisfies

(27) $${\tilde{s}}_{j}=s_{j}^{f}+G_{j}({\tilde{s}}_{j+1}-s_{j+1}^{-}),\;\;\;\;\;\;j=m_{k}-1,m_{k}-2,\ldots ,k.$$

The two-dimensional position component of the smoothed state *k* at state time ${\tilde{s}}_{k}$ is extracted as the reference $r_{k}={\left[{\tilde{x}}_{k},{\tilde{y}}_{k}\right]}^{T}$. Equations (26) and (27) are the standard RTS backward-gain and smoothed-state recursions [23].

For state time *k*, the terminal point of the RTS backward recursion is $m_{k}=\min(N,k+n)$, so the CA-RTS reference uses only the next *n* measurements and becomes available at time *k* + *n*. The corresponding output latency is $\tau_{d}$ = *nT*. This formulation makes the delay explicit and permits the lag order to be selected according to the required balance between local smoothing stability and output timeliness.

The core screening step is convex-constrained projection. The final estimate is defined as the Euclidean projection of $r_{k}$ onto the convex feasible domain of the filtering-network outputs:

(28) $${\hat{p}}_{k}^{S}=\Pi_{D_{k}}(r_{k})=arg\underset{p\in D_{k}}{min}{\left\|p-r_{k}\right\|}_{2}^{2}$$

Equivalently, it can be written as a constrained least-squares problem with respect to the convex-combination weights:

(29) $$\lambda_{k}^{*}=arg\underset{\lambda \in \Delta_{4}}{min}{\left\|\sum_{i=0}^{3}\lambda_{i}{\hat{p}}_{k}^{\left(i\right)}-r_{k}\right\|}_{2}^{2}$$

where $\Delta_{4}=\left\{\lambda \left|\sum_{i=0}^{3}\lambda_{i}=1,\lambda_{i}\geq 0\right.\right\}$ is the four-dimensional probability simplex. The screened output is given by the optimal weights:

(30) $${\hat{p}}_{k}^{S}=\sum_{i=0}^{3}\lambda_{k,i}^{*}{\hat{p}}_{k}^{\left(i\right)}$$

If the CA-RTS reference lies inside the convex hull, the projection returns the reference itself; otherwise, the solution is the nearest point on the hull boundary. Equations (28)–(30) follow directly from Euclidean projection onto a closed convex set: substituting the convex-combination representation in Equation (21) yields the simplex-constrained least-squares problem, and the optimal weights reconstruct the screened position. The projection therefore preserves continuous multi-network fusion while limiting the influence of candidates that are inconsistent with the expert set.

The convex-domain screening operation is dimension independent. With three-component filter outputs and a three-component CA-RTS reference, the feasible set becomes the convex hull of the four expert positions in three-dimensional Cartesian space. Equations (28)–(30) retain the same simplex constraints and are solved with the three-dimensional Euclidean residual. Thus, only the dimension of the candidate and reference vectors changes; the interpretation of the optimal coefficients as weights that suppress trajectory-inconsistent experts remains unchanged.

The screening operation can be stated explicitly as follows. At state time *k*, the four model-specific Bi-GRU filters independently generate the candidate positions $p_{k}^{\left(i\right)}$, which form the convex feasible domain $D_{k}$. After the next *n* measurements become available, the CA-RTS backward recursion produces a locally smoothed reference position $r_{k}$ for the same state time. The constrained least-squares problem in Equation (29) then determines nonnegative unit-sum weights whose weighted candidate position is closest to $r_{k}$. A model-mismatched candidate that departs from the locally smoothed maneuver trajectory consequently receives a small or zero coefficient, whereas candidates that remain consistent with the trajectory retain effective weights. Equation (30) fuses these retained candidates to produce the final estimate. The CA-RTS smoother therefore does not replace the network estimates; it supplies the local trajectory reference that suppresses inconsistent filter outputs during CV, CA, and CT transitions, thereby reducing transient maneuver-tracking error.

Algorithm 1 summarizes the finite-lag execution of the screened IMM-GRU filter. The four Bi-GRU filters first generate candidate positions for state time *k*. After *n* subsequent measurements have been acquired, the CA-RTS recursion computes the local reference for that same state time. The algorithm then projects the reference onto the convex domain of the four candidate positions and uses the resulting constrained weights to fuse the credible candidates.

The CA-RTS output is used solely as the screening reference for the filtering-net work candidates; it is not used by the classifier or the radar-silent prediction module. The estimate associated with state time *k* is evaluated against the ground truth at the same state time and becomes available at *k* + *n*. Hence, the screening stage changes only the availability time by *nT*; it does not change the radar sampling interval or the sequence of filtering updates. All compared methods use identical trajectories, noise realizations, sampling intervals, and state-time evaluation points, and the finite output latency of the proposed method is reported explicitly.
**Algorithm 1.** Screened IMM-GRU filtering-tracking algorithm**Input:** Radar measurement sequence $Z_{k}$, sequence length *L*, four filtering networks ${\left\{f_{i}(\cdot )\right\}}_{i=0}^{3}$, classification network C(⋅), polar-coordinate noise covariance $R_{k}$, integer fixed-lag order *n*, and CA process-noise intensity $q_{s}$.**Output:** Final position estimate ${\hat{p}}_{k}^{S}$ obtained by screened filtering.**Step 1.** For each time k, update the measurement sequence of length *L* and obtain the normalized measurement sequence ${\bar{Z}}_{k}$ by min-max normalization.**Step 2.** Input the normalized measurements into the four GRU filtering networks to obtain ${\hat{p}}_{k}^{\left(i\right)}$,i=1,2,3,4.**Step 3.** Construct the convex feasible domain $D_{k}$ of the current network from the outp-uts of the four filtering networks.**Step 4.** Convert the polar measurement into a Cartesian measurement and obtain $R_{k}^{c}$ through Jacobian propagation.**Step 5.** Perform forward Kalman filtering under the CA model to obtain $s_{k}^{f},P_{k}^{f},s_{k}^{-},P_{k}^{-}$.**Step 6.** For each state time, take $m_{k}=\min(N,k+n)$ and perform RTS backward smo-othing over the interval $\left[k,m_{k}\right]$ to obtain the reference position $r_{k}$.**Step 7.** Project $r_{k}$ onto the network convex feasible domain $D_{k}$ to obtain ${\hat{p}}_{k}^{S}=\Pi_{D_{k}}(r_{k})$.

### 3.2. Screened IMM-GRU Prediction-Tracking Algorithm

#### 3.2.1. Direct IMM Fusion Prediction Tracking

Unlike the filtering stage, the input sequence in the prediction stage is no longer guaranteed to consist entirely of actual measurements; instead, it is composed of a combination of actual measurements and historical predictions. Let $B_{k}$ denote the input sequence for the classification network at time step *k*. First, based on the current input sequence, the classification network outputs the classification probabilities for four distinct motion models: $q_{k}=C(B_{k})$. Subsequently, the propagation probabilities ${\tilde{\pi }}_{k}$ are derived from the Markov transition model:

(31) $$\Pi_{ij}=p_{s}I_{\left\{i=j\right\}}+\frac{1-p_{s}}{M-1}I_{\left\{i\neq j\right\}},\;\;\;{\tilde{\pi }}_{k}=\frac{\pi_{k-1}\Pi }{\pi_{k-1}\Pi 1}$$

where M=4, $\Pi_{ij}=P(m_{k}=j\left|m_{k-1}=i\right.)$ denotes the transition probability from mode *i* to mode *j*, $\Pi =[\Pi_{ij}]\in {\mathbb{R}}^{M\times M}$ is the transition matrix, $p_{s}$ is the mode-stay probability, and $\pi_{k-1}$ is the prior probability vector at the preceding step. Equation (31) is the standard homogeneous Markov propagation used in IMM filtering [2]. A mode-stay probability of 0.85 was selected to reflect the strong persistence expected between adjacent 0.1 s samples while retaining a total transition probability of 0.15; the remaining 0.05 is assigned to each alternative mode.

For the actual fusion probability $\pi_{k}$ in the prediction phase, a fusion of both the classification probability and the propagation probability is adopted:

(32) $$\pi_{k}=\alpha q_{k}+(1-\alpha ){\tilde{\pi }}_{k}$$

In Equation (32), *α* = 0.25 was selected on the validation set so that the propagated prior remains dominant and suppresses frame-to-frame probability jitter, while the current classifier retains sufficient weight to respond to new evidence. Let the four prediction networks be $g_{0}$, $g_{1}$, $g_{2}$, and $g_{3}$, corresponding to CV, CA, CT, and maneuver motion, respectively. They operate in parallel and output the next-step mean and diagonal position variance:

(33) $$(\mu_{k+1}^{\left(i\right)},v_{k+1}^{\left(i\right)})=g_{i}(B_{k}),\;\;\;i=0,1,2,3$$

Direct fusion does not prune the set of four networks, but directly uses $\pi_{k}$ as the mixture weight. The fused mean is obtained by probability weighting, and the fused variance is obtained by the second-moment decomposition of the mixture distribution. Therefore, it includes both the heteroscedastic output of each network and the discrete disagreement among the means of different networks:

(34) $${\hat{\mu }}_{k+1}=\sum_{i=0}^{M-1}\pi_{k}^{\left(i\right)}\mu_{k+1}^{\left(i\right)}\;{\hat{v}}_{k+1}=\sum_{i=0}^{M-1}\pi_{k}^{\left(i\right)}\left[v_{k+1}^{\left(i\right)}+(\mu_{k+1}^{\left(i\right)}-{\hat{\mu }}_{k+1})⊙(\mu_{k+1}^{\left(i\right)}-{\hat{\mu }}_{k+1})\right]$$

where ⊙ denotes the Hadamard product. Equation (34) is the law of total variance for a finite mixture: the first component is the probability-weighted within-expert variance, and the second is the between-expert disagreement. Direct IMM-GRU fusion is continuous, but a low-probability expert with a large bias can still shift the fused mean and inflate the uncertainty, thereby accelerating silent-phase risk accumulation.

#### 3.2.2. Screened Prediction Tracking

The screened prediction algorithm corrects the above limitation. Instead of using a simple maximum-probability hard decision, it jointly considers classification probability, prediction variance, inter-network disagreement, and local kinematic continuity to select a more reliable set of prediction networks.

First, probability-weighted discrete disagreement is defined. It measures the deviation of the *i*-th network from the probability-dominant network family:

(35) $$D_{i}=\frac{\sum_{j=0}^{M-1}\pi_{k}^{\left(j\right)}{\left\|\mu_{k+1}^{\left(i\right)}-\mu_{k+1}^{\left(j\right)}\right\|}_{2}^{2}}{\sum_{j=0}^{M-1}\pi_{k}^{\left(j\right)}+\varepsilon }$$

The stabilizer $\varepsilon =\;10^{-6}$ in Equation (35) prevents division by zero. It is several orders of magnitude below the normalized probability terms and therefore has no material influence on the screening score.

Second, constant-velocity extrapolation deviation is introduced. Let the last three positions in the buffer be $b_{k-2}$, $b_{k-1}$, and $b_{k}$, respectively. Then the one-step extrapolated point obtained from the most recent velocity is $2b_{k}-b_{k-1}$. The velocity-continuity deviation of the *i*-th network is defined as:

(36) $$S_{i}={\left\|\mu_{k+1}^{\left(i\right)}-(2b_{k}-b_{k-1})\right\|}_{2}$$

Equation (36) follows from one-step finite-difference extrapolation of the latest velocity. It does not assume globally constant-velocity motion; instead, it penalizes a candidate that breaks the most recent local velocity trend without supporting evidence, which is particularly useful for suppressing a mismatched maneuver expert in stable segments.

For turning or accelerating segments, velocity continuity alone is still insufficient to characterize curvature variation. Therefore, the consistency between the predicted second-order difference and the existing second-order difference in the buffer is further compared:

(37) $$A_{i}={\left\|\left(\mu_{k+1}^{\left(i\right)}-2b_{k}+b_{k-1})-(b_{k}-2b_{k-1}+b_{k-2}\right)\right\|}_{2}$$

Equation (37) compares two second-order finite differences: the acceleration surrogate implied by the candidate prediction and that observed at the end of the buffer. This term complements velocity continuity in turning or accelerating segments and penalizes candidates that introduce an abrupt, unsupported change in local curvature or acceleration.

Combining the above three quantities, the proxy uncertainty of the *i*-th network is defined as:

(38) $$u_{i}=\lambda_{d}D_{i}+\lambda_{s}S_{i}+\lambda_{a}A_{i}$$

The coefficients $\lambda_{d}$ = 0.001, $\lambda_{s}$ = 0.5, and $\lambda_{a}$ = 0.5 in Equation (38) were selected on the independent validation set after accounting for term scale. Pairwise disagreement aggregates all experts and is numerically larger, so its coefficient is reduced; velocity and second-order consistency receive equal weight as complementary local kinematic constraints. The values are fixed before testing.

Probability gating is then performed. The model probabilities are sorted in descending order to obtain the index sequence $I_{k}=(i_{1},i_{2},\cdots ,i_{M})$, satisfying:

(39) $$\pi_{k}^{\left(i_{1}\right)}\geq \pi_{k}^{\left(i_{2}\right)}\geq \ldots \geq \pi_{k}^{\left(i_{M}\right)}$$

Using the descending order in Equation (39), the minimum number of networks whose cumulative probability reaches the posterior-mass threshold is defined as follows:

(40) $$r_{k}=min\left\{r:\sum_{l=1}^{r}\pi_{k}^{\left(i_{l}\right)}\geq \tau_{post}\right\}$$

The validation-selected threshold $\tau_{post}$ = 0.85 in Equation (40) retains the smallest subset carrying most of the posterior support, so low-probability experts are removed before kinematic scoring. To avoid both single-model brittleness and four-model dilution, the retained-set size is constrained by $m_{\min}$ and $m_{\max}$:

(41) $$m_{k}=\min\{m_{\max},\max\{m_{\min},r_{k}\}\}$$

The bounds $m_{\min}$ = 2 and $m_{\max}$ = 3 preserve one alternative motion hypothesis while excluding at least one weak expert. Near a mode transition, posterior ambiguity is then assessed using normalized entropy and the top-two probability margin:

(42) $$H_{k}=-\frac{1}{\ln M}\sum_{i=0}^{M-1}\pi_{k}^{\left(i\right)}\ln(\pi_{k}^{\left(i\right)}+\varepsilon )\;\Delta_{k}=\pi_{k}^{\left(i_{1}\right)}-\pi_{k}^{\left(i_{2}\right)}$$

The term $\varepsilon =\;10^{-6}$ in Equation (42) prevents log(0) and is negligible on the probability scale. Division by ln*M* normalizes the entropy to [0, 1]. A large entropy or small top-two margin indicates ambiguity, in which case the maneuver expert is retained:

(43) $$R_{k}=\left\{\begin{array}{cc} R_{k}^{0}\cup \{i_{man}\}, & H_{k}>\;\tau_{H}\vee \Delta_{k}<\;\tau_{\Delta },\\ R_{k}^{0}, & H_{k}\leq \tau_{H}\wedge \Delta_{k}\geq \tau_{\Delta }. \end{array}\right.$$

The validation-selected thresholds $\tau_{H}$ = 0.75 and $\tau_{\Delta }$ = 0.10 identify, respectively, a diffuse posterior and a near tie between the leading modes. Either condition retains the maneuver expert, protecting transition intervals without activating it when the classifier is decisive.

Within the candidate set $R_{k}$, a logarithmic score is further used to measure the overall reliability of each network:

(44) $$J_{i}=\ln u_{i}-\lambda_{p}\ln(\pi_{k}^{\left(i\right)}+\varepsilon ),\;\;\;i\in R_{k}.$$

In Equation (44), $\lambda_{p}$ = 0.2 was selected on the validation set so that kinematic inconsistency remains the primary criterion and posterior probability acts as a secondary preference. The term $\varepsilon =\;10^{-6}$ provides the same numerical safeguard; both values are fixed before testing.

The candidate networks are sorted in ascending order of $\;J_{i}$, yielding the index sequence $\left(\kappa_{1},\kappa_{2},\ldots \right)$. Finally, only the first $K_{f}$ networks are retained for fusion; this paper takes $K_{f}=2$. The final prediction-network set is therefore $S_{k}=\{\kappa_{1},\kappa_{2},\ldots ,\kappa_{K_{f}}\}$. For the networks in the final set $S_{k}$, the unnormalized weights are first constructed as:

(45) $$w_{i}=\frac{\pi_{k}^{\left(i\right)}}{u_{i}},\;\;\;i\in S_{k}.$$

The weights are then normalized within the screened set:

(46) $$w_{i}=\frac{{\tilde{w}}_{i}}{\sum_{j\in S_{k}}{\tilde{w}}_{j}},\;\;\;i\in S_{k}.$$

The final retained-set size $K_{f}=2$ was selected to preserve one alternative motion hypothesis without diluting the fusion with a third weak candidate. This avoids single-expert brittleness while retaining the screening benefit; the selected pair is then weighted by posterior probability and inverse proxy uncertainty.

The screened fused mean is

(47) $$\mu_{k+1}=\sum_{i\in S_{k}}w_{i}\mu_{k+1}^{\left(i\right)}$$

The fused variance still uses the second-moment decomposition of the mixture distribution, so that it reflects both the network’s own variance and the residual disagreement among the retained networks:

(48) $$v_{k+1}=\sum_{i\in S_{k}}w_{i}\left\{v_{k+1}^{\left(i\right)}+\left[\mu_{k+1}^{\left(i\right)}-\mu_{k+1}\right]⊙\left[\mu_{k+1}^{\left(i\right)}-\mu_{k+1}\right]\right\}$$

Equations (47) and (48) are the screened-set counterparts of the mixture mean and total-variance decomposition in Equation (34). After each step, the fused mean, variance trace, real-measurement flag, and prediction age are written back to the buffer. This recursion matches the multi-step rollout used in training and reduces the distribution mismatch between training and radar-silent inference.

The screened prediction algorithm extends to three dimensions by replacing every position, velocity, and second-order-difference vector in Equations (35)–(48) with its three-component counterpart. The disagreement and motion-consistency terms retain their Euclidean-norm definitions, and the probability, entropy, and candidate-set rules are unchanged. The retained experts produce three-component means and diagonal three-axis variances; the within-expert uncertainty and between-expert disagreement are combined componentwise, and the trace of the resulting three-dimensional position covariance is written back to the buffer. The screening coefficients and decision thresholds should be fixed on an independent three-dimensional validation set before testing, because spatial climb and coupled turn-climb motion can change the numerical scales of the kinematic-consistency terms.

Therefore, screened prediction does not destroy the IMM multi-model structure; rather, it removes network outputs that are incompatible with both the current posterior probability and local kinematics before fusion. This mechanism suppresses maneuver-network disturbances in stable segments and retains the maneuver network in switching segments, thereby reducing the accumulation rate of silent-prediction error.

Algorithm 2 gives the online recursion of the Screened IMM-GRU prediction. It first updates the mode probabilities and generates predictions from the four networks in parallel, then screens reliable networks according to probability, uncertainty, and kinematic consistency, and finally completes mean fusion, variance matching, and buffer write-back to obtain the multi-step predicted trajectory and its uncertainty.
**Algorithm 2.** Screened IMM-GRU prediction-tracking algorithm**Input:** Prediction input sequence $B_{k}$, previous-time model probability $\pi_{k-1}$, four pred-iction networks ${\left\{g_{i}(\cdot )\right\}}_{i=0}^{3}$, classification network C(⋅), model transition matrix Π, and screening parameter set Ω.**Output:** The predicted position ${\hat{p}}_{k+1}$ and predicted variance ${\hat{\Sigma }}_{k+1}$ at the next time step.**Step 1.** The classification network gives the current probability $q_{k}=C(B_{k})$, and $\pi_{k-1}$, Π is used to propagate the previous-time probability to obtain the fused probability $\pi_{k}$.**Step 2.** Call the four prediction networks to obtain $\mu_{k+1}^{i}$ and $v_{k+1}^{i}$, i=1,2,3,4.**Step 3.** For $B_{k}$, take its last three positions $b_{k-2}$$,\;b_{k-1}$, and $b_{k}$, calculate $D_{i}$$,\;S_{i}$$,\;\mathrm{and}\;A_{i}$, and further obtain the proxy uncertainty $u_{i}$.**Step 4.** Sort the networks according to descending $\pi_{k}^{i}$, and when the cumulative probability reaches $\tau_{\mathrm{post}}$, construct the initial candidate network set $R_{k}^{0}$; if the classification entropy $H_{k}$ is too large or the gap between the two largest probabilities Δπ is too small, additionally retain the maneuver network to obtain the final candidate network set $R_{k}$.**Step 5.** Compute the score $u_{i}$ within the candidate set $R_{k}$, and select the $K_{f}$ networks with the smallest scores to form $S_{k}$.**Step 6.** Within $S_{k}$, compute the normalized weights $w_{i}$, and complete mean fusion and variance moment matching.**Step 7.** Write the fused mean, real flag 0, variance trace, and incremented age back to the buffer; update the model probability and record the prediction result.**Step 8.** Output the predicted position and predicted variance.

## 4. Radar Low-Probability-of-Intercept Tracking Algorithm Jointly Driven by Networks and Models

Motivated by the LPI tracking requirement discussed in Section 1, this section develops a closed-loop radar radiation scheduling algorithm jointly driven by networks and models.

During initialization, the radar first uses the initial measurements to establish an input buffer of length *L*. After scheduling formally starts, the radar defaults to the non-radiation prediction state rather than maintaining radiation first. This treatment conforms to the basic principle of LPI tracking: as long as the prediction risk remains controlled, unnecessary active radiation is avoided; only when the prediction result is insufficient to constrain the track is radiation restored to obtain a real measurement.

In the non-radiation state, the radar does not acquire measurements. Instead, the proposed Screened IMM-GRU prediction algorithm outputs the next-step position mean and variance, and writes the predicted point back to the sliding window. Because continuous prediction gradually weakens the constraint of real measurements on the track, this paper accumulates the trace of the fused variance output by screened prediction within the current non-radiation interval as the criterion for restoring radiation:

(49) $$U_{k+1}=U_{k}+tr({\hat{\Sigma }}_{k+1}),\;U_{k_{0}}=0$$

where tr(⋅) is the trace operator and $k_{0}$ is the first sample of the current silent interval. Equation (49) accumulates the screened position-variance trace over that interval. The resulting nondecreasing statistic acts as a prediction-risk variable: once it reaches the radiation-restoration threshold, the radar resumes transmission and obtains a new measurement.

After radiation is restored, the radar writes measurements into the sliding window and performs filtering using the proposed Screened IMM-GRU filtering algorithm. At this point, the scheduling objective is not to prolong the radiation duration, but to correct the prediction error accumulated during the non-radiation phase as quickly as possible using real measurements. To determine whether the track has recovered to a reliable state, this paper adopts the normalized innovation squared as the measurement-consistency metric:

(50) $${\mathrm{NIS}}_{k}={\left(z_{k}-h({\hat{p}}_{k}^{S})\right)}^{T}S_{k}^{-1}(z_{k}-h({\hat{p}}_{k}^{S}))$$

When the NIS falls below the radiation-termination threshold, the filtered state is sufficiently consistent with the current measurement and the radar returns to silent prediction. Equation (50) is the standard normalized innovation squared [27]; scaling the residual by its predicted covariance provides a dimensionless consistency test rather than an unnormalized distance.

For three-dimensional operation, the radiation-restoration statistic in Equation (49) uses the trace of the three-dimensional position covariance. The innovation vector in Equation (50) contains the Cartesian x-, y-, and z-residuals, and its covariance includes the transformed range-azimuth-elevation measurement uncertainty. The NIS therefore follows a three-degree-of-freedom consistency test under the nominal Gaussian model, so the radiation-termination threshold must be recalibrated accordingly. Apart from these covariance and threshold changes, the silent-prediction, radiation-restoration, screened-correction, and radiation-termination state logic is unchanged.

**Figure 5:**
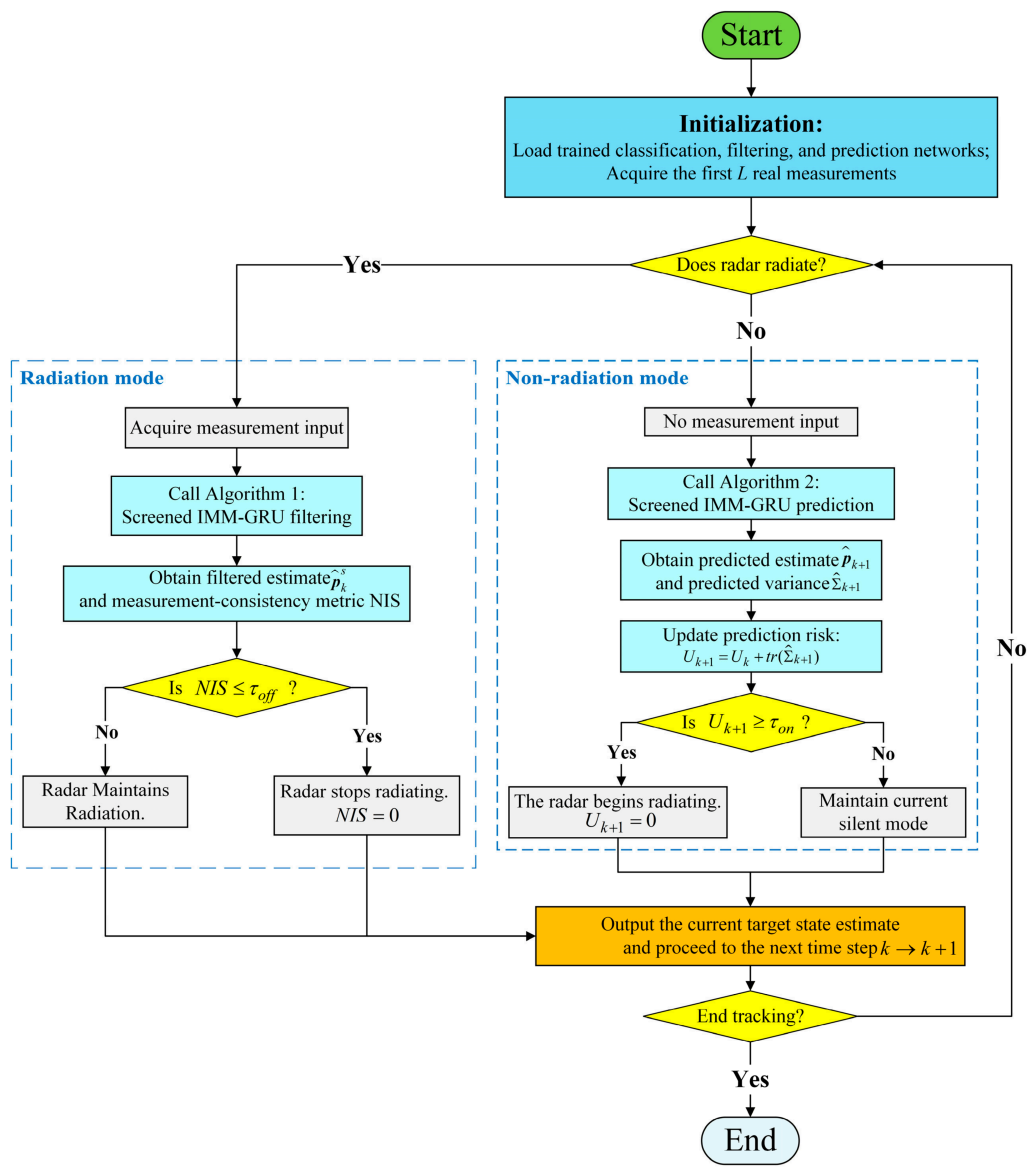
Flowchart of the radar LPI tracking algorithm jointly driven by networks and models.

Figure 5 summarizes the closed-loop LPI tracking process jointly driven by networks and models. In the non-radiation phase, Screened IMM-GRU prediction maintains the track and uses the accumulated prediction variance to determine whether radiation should be restored; in the radiation phase, real measurements and Screened IMM-GRU filtering are used to correct the state, and NIS is used to determine whether the radar should be turned off again, thereby enabling dynamic switching between tracking accuracy and low-radiation requirements.

## 5. Simulation Analysis

### 5.1. Network Training Settings

The training trajectories are generated from the kinematic models described above, with independently sampled initial states, process noise, and measurement noise. Each input contains *L* = 100 samples at *T* = 0.1 s and therefore spans 10 s. Maneuver windows place a model transition within the first or last 3 s, so this length retains the transition and at least 7 s of steady motion on one side, providing the stable reference needed to estimate velocity, curvature, and pre-/post-transition consistency. Shorter windows remove this reference segment, whereas longer windows mainly add older, less relevant states and increase recurrent cost [3,8]. Thus, *L* = 100 is fixed for training, validation, testing, and all temporal baselines as a problem-specific choice rather than a universal optimum. The remaining data-generation parameters are listed in Table 1.

**Figure 6:**
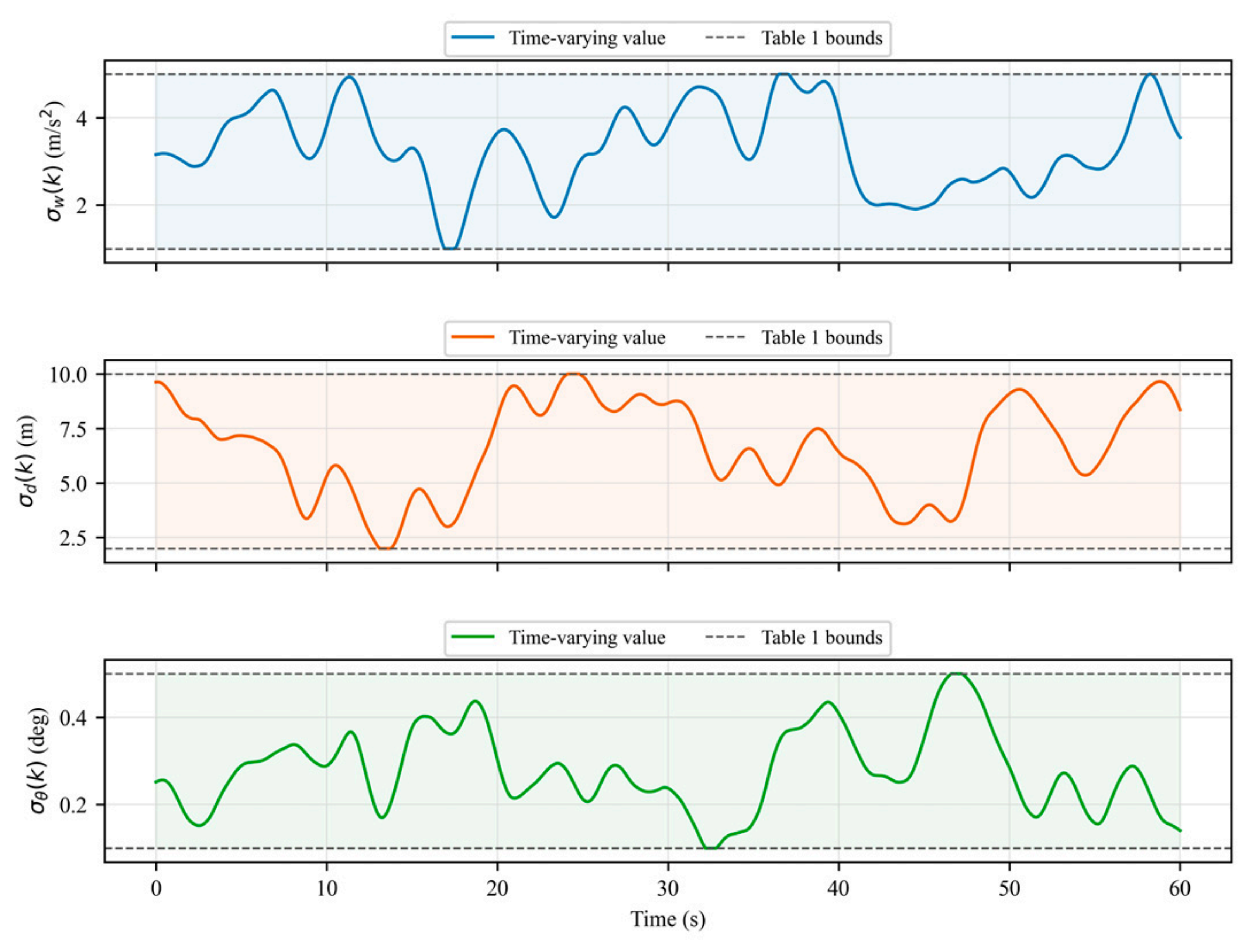
Representative time-varying standard-deviation profiles used in the simulations for process noise, range measurement noise, and bearing measurement noise. Dashed lines indicate the corresponding bounds in Table 1.

To represent time-varying sensing conditions, the process-noise, range-noise, and bearing-noise standard deviations are resampled at every time step within the intervals specified in Table 1. Conditional on these instantaneous standard deviations, the corresponding samples are drawn from zero-mean Gaussian distributions. Figure 6 shows one representative realization of the three standard-deviation sequences, with dashed lines indicating the prescribed lower and upper bounds. The simulations therefore use time-dependent noise variances while keeping all instantaneous values within the ranges encountered during network training.

The filtering networks are trained separately according to motion modes. The CV, CA, and CT filtering networks each use 300,000 single-model samples, while the maneuver filtering network uses 900,000 model-switching samples. The training and validation sets are divided at a ratio of 8:2.

The maximum rollout horizon is *H* = 30, corresponding to 3 s at *T* = 0.1 s. This value matches the longest measurement-free horizon evaluated and ensures that the predictors are trained across the complete transition interval rather than only for one-step extrapolation. Each sample therefore contains a 100-point history and 30 future targets. The CV, CA, and CT predictors each use 300,000 samples, and the maneuver predictor uses 600,000 samples.

The classification network and the filtering networks use the same trajectory sources and are labeled according to the true motion mode within the window. To enhance switching recognition, most windows in the maneuver samples are set to contain model transitions within the first 3 s or the last 3 s of the window.

Table 2 lists the main training parameters. All networks are optimized using Adam. The learning rates of the filtering and classification networks are decayed by a factor of 0.1 every 10 epochs, and the learning rate of the maneuver prediction network is decayed by a factor of 0.3 every 10 epochs.

### 5.2. Simulation Parameter Settings

#### 5.2.1. Simulation Scenarios

Four representative horizontal-plane maneuvering scenarios are used to evaluate the algorithms, as summarized in Table 3. The training and test trajectories are generated independently with disjoint random seeds. Their initial conditions, maneuver-transition times, and time-varying noise realizations differ, although both sets remain within the CV/CA/CT model families and physical parameter ranges in Table 1. The reported results therefore assess robustness to independently sampled in-distribution trajectories, rather than to unmodeled dynamics or real-radar domain shift. Validation under those broader conditions is reserved for future measured-data studies.

#### 5.2.2. Comparison Algorithms

The comparison set is designed to cover single-model and multi-model estimators, model-driven and data-driven methods, and both measurement-updated filtering and measurement-free prediction. It includes EKF, IMM-EKF, GRU, direct IMM-GRU, the adapted IMM-EKF-CNN-LSTM hybrid proposed in [28], and the IMM future-state predictor of Wells et al. [29].

EKF filtering algorithm: the CV model is adopted as the reference state-transition model.

IMM-EKF filtering algorithm: the candidate models include a CV model, a CA model with an acceleration magnitude of 15 m/s^2^, and CT models with turn rates of +/−10°/s. The stay probability in the model transition matrix is set to 0.8.

Adapted IMM-EKF-CNN-LSTM filter [28]: following the hybrid state-estimation and residual-compensation principle of She et al., IMM-EKF first produces a nominal position sequence. At each sample, the two-dimensional measurement, the IMM-EKF position, and their innovation are concatenated into a six-dimensional feature. A 100-sample feature window is processed by two one-dimensional convolutional layers (48 channels, kernel size 5; 64 channels, kernel size 3), followed by a two-layer LSTM with 96 hidden units and a 96-64-2 fully connected residual head. The predicted two-dimensional residual is added to the current IMM-EKF position. For the present implementation, 6000 independently generated trajectories are used for training and 1200 for validation. AdamW is run for 18 epochs with batch size 192, initial learning rate 10^−3^, and weight decay 10^−5^; the learning rate is reduced on a validation plateau, and the checkpoint with the minimum validation loss is retained for all tests.

EKF prediction algorithm: the CV model is also adopted. This algorithm first uses the first 100 polar measurements for filtering initialization, and then performs only state prediction without measurement updates.

IMM-EKF prediction algorithm: the same candidate models and parameters as the IMM-EKF filtering algorithm are used. This algorithm first uses the first 100 polar measurements for initialization, and then performs only IMM state prediction without introducing new measurement corrections.

Wells et al. IMM future-state predictor [29]: after the final measurement update, the CV, CA, and CT branches provide model-conditioned states and posterior probabilities. Their probability-weighted mixture defines the current state, while the branch with the largest posterior probability supplies the maximum-a-posteriori motion hypothesis used for future propagation. Its transition matrix is then applied recursively over the common 30-step measurement-free horizon. This model-driven baseline requires no network training. Only the state interface is adapted to the present horizontal-plane problem; initialization, noise realizations, maneuver windows, and evaluation metrics are identical to those of the other predictors. Unlike the standard IMM-EKF predictor, the Wells-type method does not continue model interaction and probability-weighted state fusion throughout the rollout.

The initial states of all traditional filters are set to the true initial target states, and the initial covariance is the identity matrix. Because the GRU-based methods require a 100-sample (10 s) history window, performance statistics begin after the initialization interval.

#### 5.2.3. Simulation Performance Evaluation Metrics

The two-dimensional position error is used as the core evaluation metric. For filtering and LPI tracking, the multi-Monte-Carlo mean root mean square error (MRMSE) is used to evaluate full-period accuracy. For the prediction networks, the prediction-horizon RMSE is further used to characterize error growth with extrapolation length.

For the *r*-th Monte Carlo run, let the position error at time *k* be *e*^(*r*)^. All reported filtering and prediction experiments use *N*_MC_ = 500 runs, which was chosen to suppress run-to-run fluctuation in the reported MRMSE while keeping the simulation tractable. The time-resolved position RMSE is

(51) $$\mathrm{RMSE}(k)=\sqrt{\frac{1}{N_{\mathrm{MC}}}\sum_{r=1}^{N_{\mathrm{MC}}}{\left\|{\hat{p}}_{k}^{\left(r\right)}-p_{k}^{\left(r\right)}\right\|}_{2}^{2}}$$

Equation (51) computes the Euclidean position RMSE across Monte Carlo runs at each time instant. To summarize performance over both time and runs, the overall MRMSE is

(52) $$\mathrm{MRMSE}=\sqrt{\frac{1}{N_{\mathrm{MC}}K}\sum_{r=1}^{N_{\mathrm{MC}}}\sum_{k=1}^{K}{\left\|{\hat{p}}_{k}^{\left(r\right)}-p_{k}^{\left(r\right)}\right\|}_{2}^{2}}$$

Equation (52) averages the squared Euclidean position error jointly over evaluation times and Monte Carlo runs before taking the square root. For measurement-free prediction, error growth is also evaluated at each forecast horizon:

(53) $${\mathrm{RMSE}}_{\mathrm{pred}}(h)=\sqrt{\frac{1}{N_{\mathrm{MC}}}\sum_{r=1}^{N_{\mathrm{MC}}}{\left\|{\hat{p}}_{k_{0}+h}^{\left(r\right)}-p_{k_{0}+h}^{\left(r\right)}\right\|}_{2}^{2}},\;\;\;\;\;\;h=1,2,\ldots ,H.$$

Equation (53) applies the same Monte Carlo averaging at each prediction step h. Equations (51)–(53) are used without modification for every comparison method.

### 5.3. Performance Verification of the Three GRU Networks

This section verifies the three basic networks in sequence: the ability of the classification network to discriminate motion models, the noisy-window smoothing ability of the filtering network, and the short-term measurement-free extrapolation ability of the prediction network.

#### 5.3.1. Classification Network

Classification-network performance is evaluated according to the ground-truth motion-model intervals defined in Table 3. In each scenario, the dominant output probability is expected to correspond to the true CV, CA, or CT segment, while the maneuver-class probability should respond to model-transition intervals.

**Figure 7:**
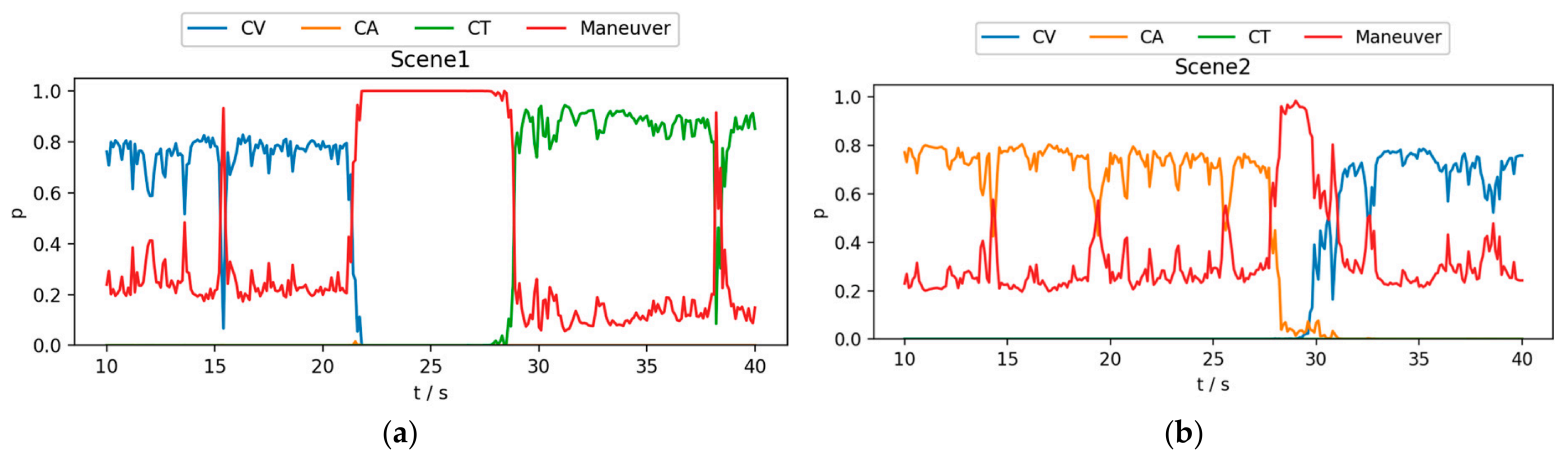

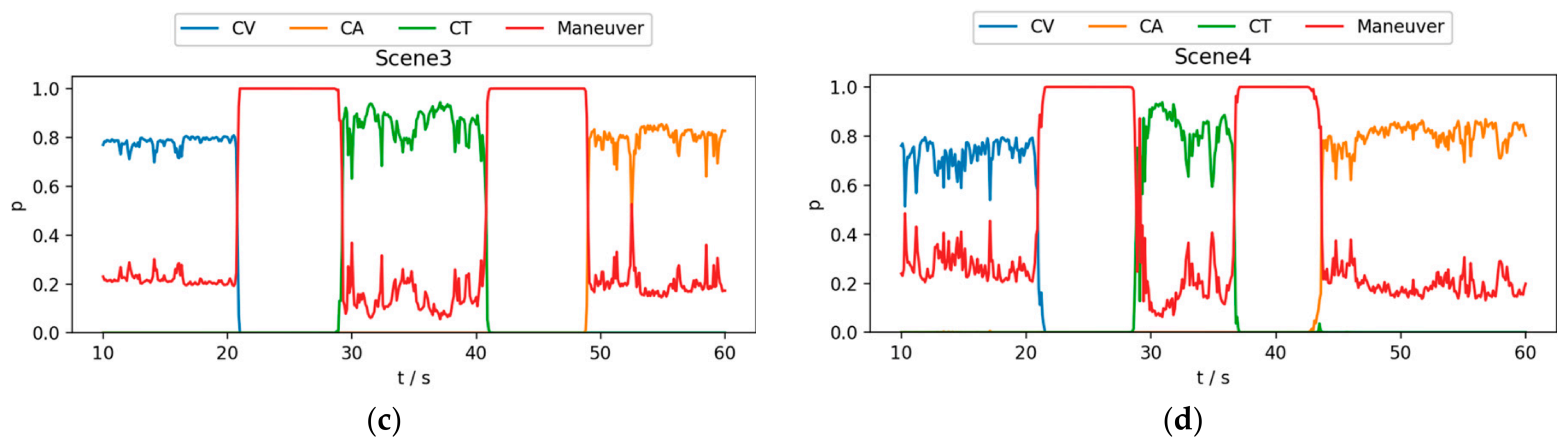
Model-probability outputs of the classification network in four simulation scenarios. (**a**) Scenario 1; (**b**) Scenario 2; (**c**) Scenario 3; (**d**) Scenario 4.

Figure 7 shows the temporal probability outputs of the classification network in the four simulation scenarios. The dominant probabilities generally agree with the motion models specified in Table 3: CV, CA, and CT probabilities are dominant in their corresponding stable-motion intervals, whereas the maneuver probability rises rapidly near model-switching intervals. In particular, Scenes 3 and 4 contain two model transitions, and the maneuver-class probability exhibits two corresponding high-response regions. These results indicate that the classification network can distinguish stable motion modes and capture transition states, thereby providing reliable mode priors for the subsequent Screened IMM-GRU filtering and prediction algorithms.

#### 5.3.2. Filtering Network

Single-network filtering performance is evaluated by comparing GRU-CV with EKF-CV in all four scenarios; the resulting MRMSE values are listed in Table 4.

**Figure 8:**
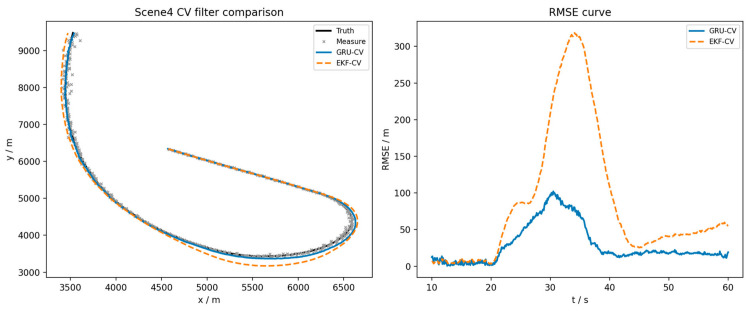
Comparison of tracking results of GRU-CV and EKF-CV in Scenario 4.

Table 4 shows that GRU-CV is significantly superior to EKF-CV in all four scenarios, indicating that the filtering network can use the overall geometric shape of historical measurement windows to perform nonlinear sequence smoothing. Figure 8 provides the trajectory and RMSE comparison in Scenario 4.

However, a single CV network cannot cover strong CT or CA maneuvering segments. Therefore, CA, CT, and maneuver networks still need to be introduced, and multi-model fusion must be completed through classification probabilities and the screening mechanism.

#### 5.3.3. Prediction Network

**Figure 9:**
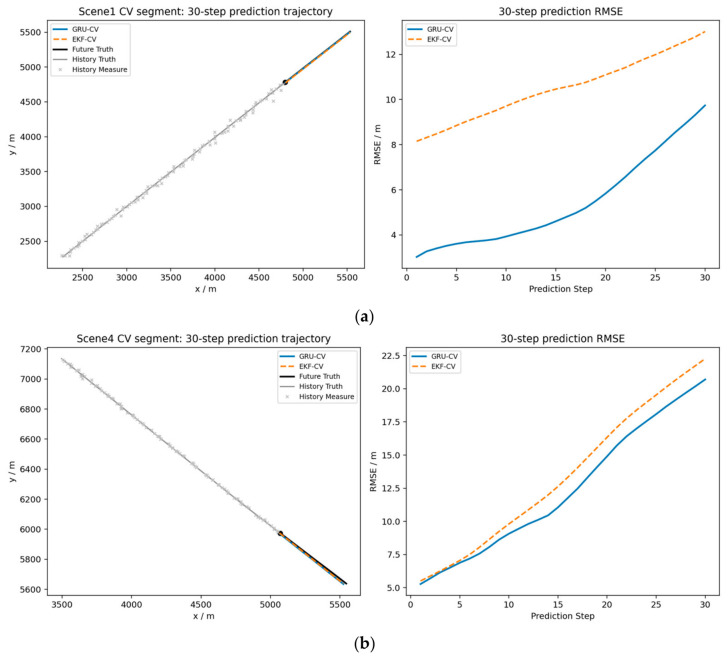
Prediction-performance comparison between GRU-CV and EKF-CV in CV segments. (**a**) Scenario 1; (**b**) Scenario 4.

Single-prediction-network validation compares the GRU-CV and EKF-CV prediction algorithms. Both algorithms are initialized using the first 10 s of measurements and then perform 30-step extrapolation without new measurements. The CV segments from Scenarios 1 and 4 are selected to evaluate the short-term silent prediction performance of GRU-CV and EKF-CV, as shown in Figure 9.

As shown in Figure 9, GRU-CV achieves lower 30-step prediction RMSE than EKF-CV in both selected CV segments. The reduction is more evident in Scenario 1, reaching about 25%, because the GRU predictor can better exploit the recent trajectory pattern and suppress error accumulation during long-horizon silent extrapolation. In Scenario 4, the improvement is relatively moderate, about 7%, since the selected segment is well matched by the CV assumption of EKF-CV. Nevertheless, GRU-CV still maintains lower prediction error, confirming its advantage in radar-silent track maintenance.

In summary, the classification, filtering, and prediction networks respectively provide mode-probability priors, noisy-window smoothing, and short-term silent extrapolation, forming the foundation for subsequent Screened IMM-GRU filtering and prediction as well as LPI tracking.

### 5.4. Performance Analysis of IMM Filtering and Prediction

#### 5.4.1. IMM-GRU Filtering-Tracking Simulation

To evaluate the screened filtering strategy in Section 3.1.2, four methods are tested under the same trajectories and noise realizations: IMM-EKF, the adapted IMM-EKF-CNN-LSTM hybrid [28], direct IMM-GRU, and the proposed screened IMM-GRU.

**Figure 10:**
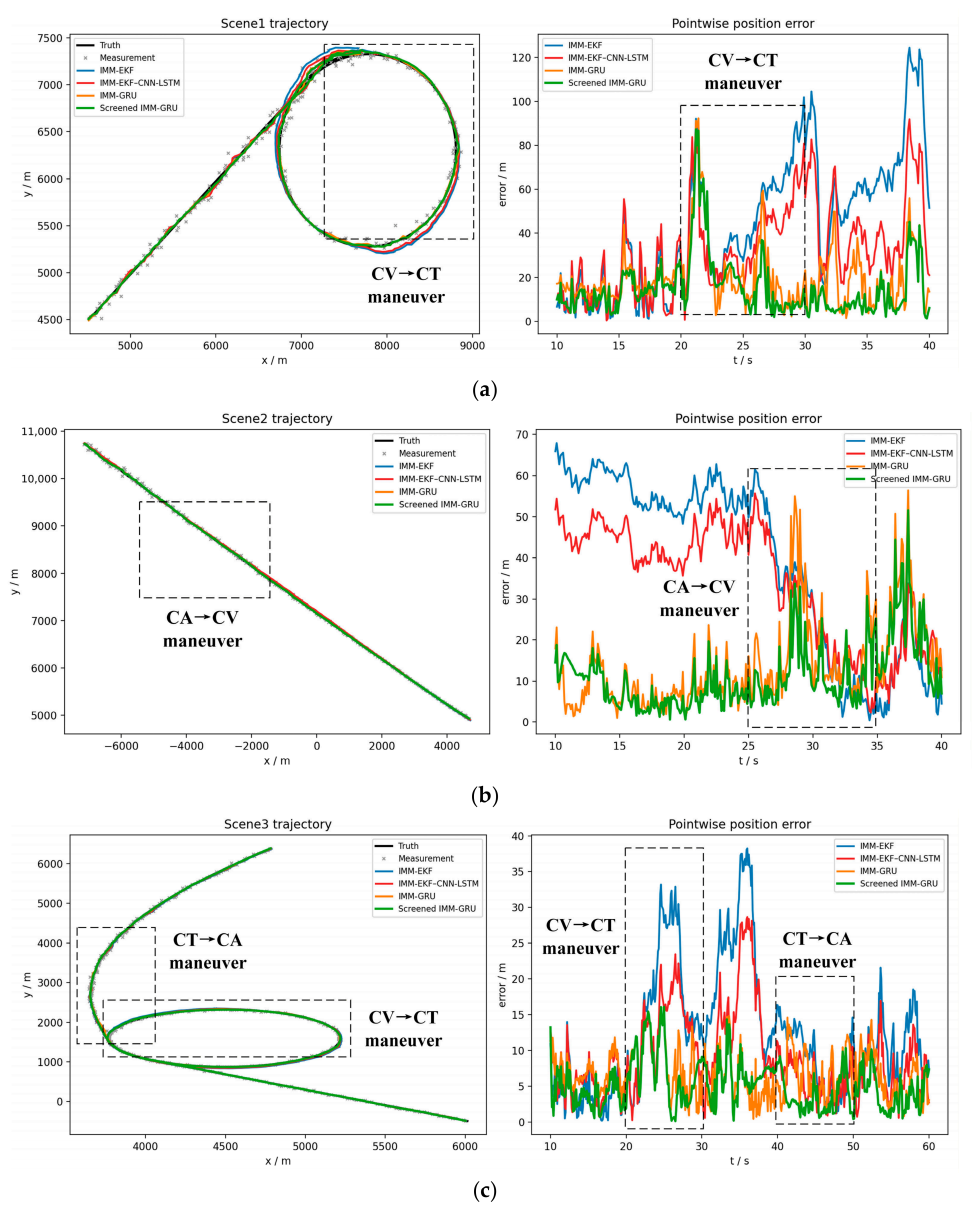

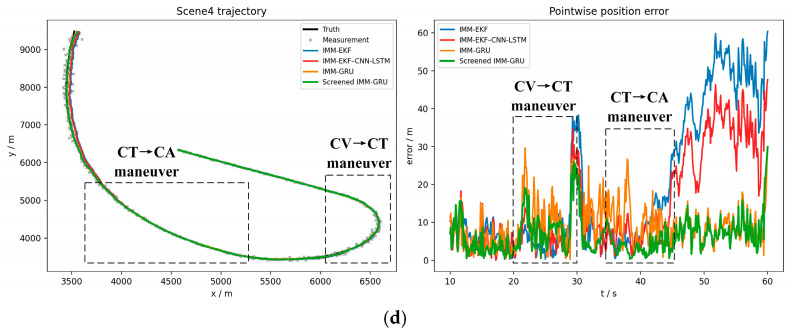
Tracking performance of IMM filtering algorithms in four scenarios. (**a**) Scenario 1; (**b**) Scenario 2; (**c**) Scenario 3; (**d**) Scenario 4.

For the filtering experiments, the fixed-lag order is restricted to *n* ∈ {1, 2, 3}. With the sampling interval *T* = 0.1 s, the corresponding output latency *nT* is 0.1–0.3 s. The numerical results in Figure 10 and Table 5 and Table 6 use *n* = 3, the most conservative setting in this range. Thus, each screened estimate uses at most three subsequent measurements and is available no later than 0.3 s after the associated state time. This setting provides the local smoothing information required to identify filter outputs that are inconsistent with the maneuver trajectory while retaining a bounded sub-second latency.

Figure 10 shows that the adapted IMM-EKF-CNN-LSTM method generally improves upon IMM-EKF by compensating persistent model-induced bias, but larger transients remain around mode changes. Direct IMM-GRU further reduces these deviations through mode-specific nonlinear sequence estimation. Across the four representative runs, the screened IMM-GRU trajectory remains closest to the ground truth overall and its pointwise position-error curve is generally the lowest, consistent with the Monte Carlo summaries in Table 5 and Table 6.

Table 5 confirms the same ordering over the complete trajectories. Relative to IMM-EKF, the adapted IMM-EKF-CNN-LSTM baseline lowers the MRMSE from 48.71, 44.37, 16.40, and 25.35 m to 32.31, 36.56, 12.31, and 18.18 m in Scenarios 1–4, demonstrating the value of learned residual compensation. Direct IMM-GRU performs better still, while screened IMM-GRU achieves the minimum MRMSE in every scenario: 16.62, 14.24, 7.43, and 8.08 m. These values are 17.31–26.55% lower than those of direct IMM-GRU, showing that the gain is attributable not merely to adding a neural corrector to IMM-EKF, but to mode-specific network estimation followed by the screening of inconsistent candidates.

Table 6 focuses on the six maneuver-transition intervals. The adapted IMM-EKF-CNN-LSTM baseline improves upon IMM-EKF in every interval and also outperforms direct IMM-GRU in the Scenario 3 CT-to-CA segment and both Scenario 4 segments. Nevertheless, screened IMM-GRU obtains the lowest MRMSE in all six cases. This result indicates that a single residual-correction branch can mitigate systematic IMM-EKF bias, whereas abrupt transitions still benefit from the proposed convex-hull screening, which rejects candidate estimates that are incompatible with the current multi-network feasible domain.

#### 5.4.2. IMM-GRU Prediction-Performance Simulation

To evaluate the screened prediction strategy in Section 3.2.2, four predictors are tested on the same six maneuver windows and the same 30-step horizon: standard IMM-EKF prediction, the maximum-posterior IMM future-state predictor adapted from Wells et al. [29], direct IMM-GRU, and screened IMM-GRU.

Table 7 shows that the Wells-type predictor improves upon standard IMM-EKF in five of the six maneuver windows, confirming the usefulness of maximum-posterior model propagation when the dominant mode is identified reliably. Direct IMM-GRU is more accurate than both model-driven predictors in every segment, indicating that the recurrent networks better represent nonlinear evolution across mode transitions. Screened IMM-GRU achieves the lowest MRMSE in all six segments. Relative to direct IMM-GRU, the reductions are 59.38%, 83.50%, 14.64%, 13.70%, 11.60%, and 50.27%, respectively, demonstrating that the improvement is consistent across transition types rather than being driven by a single favorable case.

**Figure 11:**
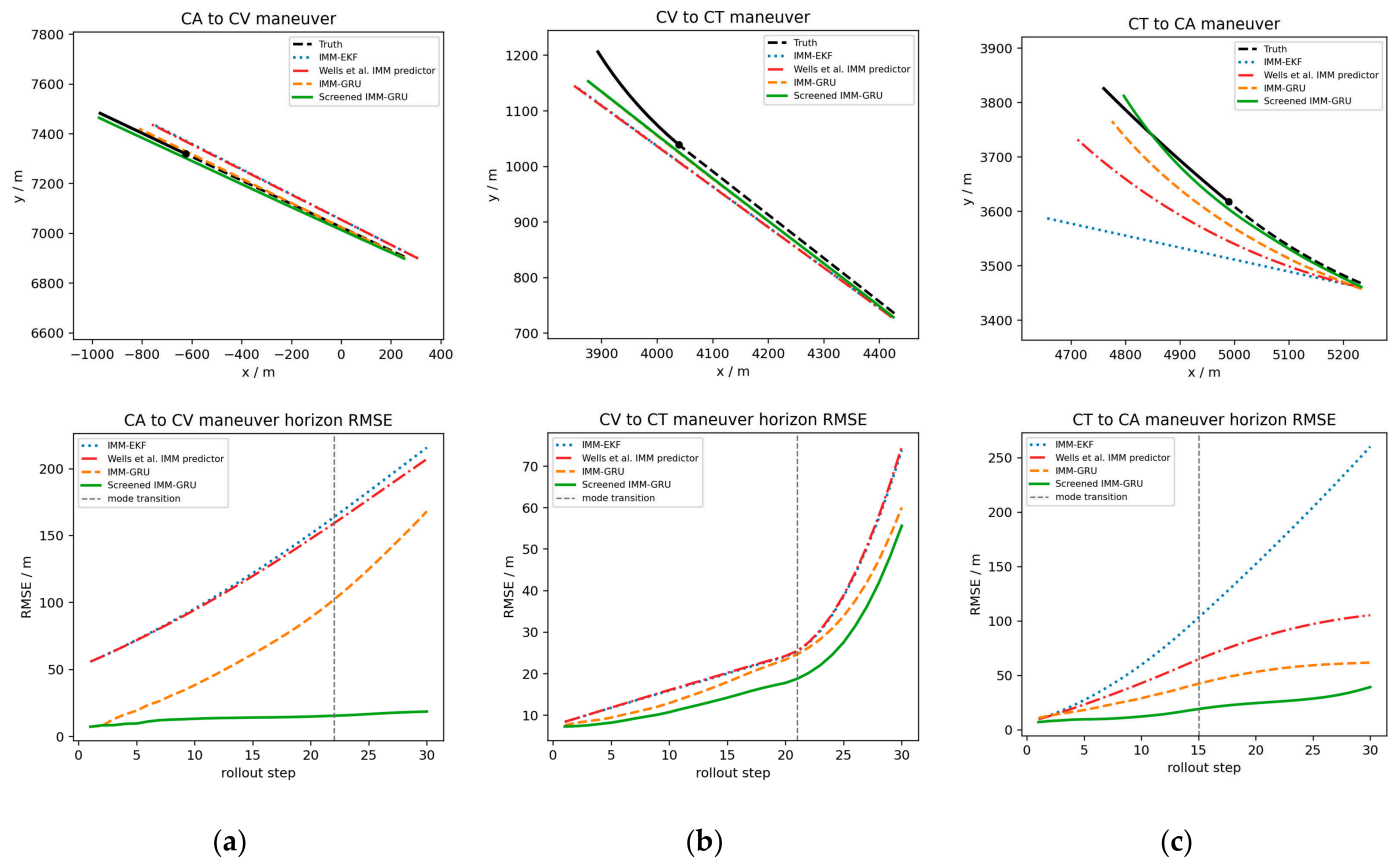
Prediction trajectories and raw horizon RMSE curves of IMM-EKF, the Wells et al. IMM predictor, IMM-GRU, and Screened IMM-GRU for three representative maneuvering segments. The black point and vertical dashed line denote the true motion-mode transition. (**a**) CA-to-CV maneuver; (**b**) CV-to-CT maneuver; (**c**) CT-to-CA maneuver.

Figure 11 complements the segment-level MRMSE results with representative 30-step CA-to-CV, CV-to-CT, and CT-to-CA prediction windows. In each subfigure, the black point in the trajectory panel and the vertical dashed line in the error panel mark the true mode-transition instant. Identical plotting dimensions are used to facilitate direct visual comparison among the four predictors.

Figure 11 resolves the comparison along the prediction horizon. The model-driven predictors accumulate larger errors when the retained model becomes inconsistent with the post-transition motion. In the CA-to-CV example, maximum-posterior projection provides a modest improvement over standard IMM-EKF, whereas screened IMM-GRU maintains the smallest overall error. In the CV-to-CT window, the screened curve remains below all three alternatives and separates further after the transition; its segment MRMSE is 22.39 m, compared with 26.23 m for direct IMM-GRU and 30.65–30.93 m for the model-driven predictors. The same pointwise advantage is observed in the CT-to-CA example. The figure therefore shows that multi-cue screening limits both model-mismatch error and unreliable recurrent extrapolation as the silent horizon increases.

The preceding comparison establishes the overall prediction advantage but does not isolate the contribution of each screening cue. A one-component-at-a-time ablation is therefore conducted on the same six maneuver windows used in Table 7. All variants use identical trajectories, noise realizations, trained network weights, random seeds, and 30-step horizons; only the specified screening component is removed. Performance differences can therefore be attributed to the omitted component rather than to changes in data or model capacity.

Table 8 shows that every incomplete variant performs worse than the full method in every reported segment. Averaged over the six windows, screened IMM-GRU attains an MRMSE of 22.33 m, corresponding to reductions of 48.59%, 80.41%, 43.87%, 48.55%, and 49.84% relative to the variants without model-probability gating, prediction uncertainty, inter-network disagreement, velocity continuity, and second-order-difference consistency, respectively. The largest degradation occurs when prediction uncertainty is removed, demonstrating its central role in suppressing unreliable recursive outputs. Probability gating restricts fusion to dynamically plausible experts, whereas the three consistency terms reject predictions that conflict with the network ensemble, recent velocity, or local acceleration evolution. Their complementary action explains why the complete screening rule consistently outperforms every one-component-removed alternative.

### 5.5. LPI Tracking Simulation

This section evaluates LPI efficiency under an equal-accuracy criterion. Rather than fixing the radiation budget and comparing the resulting tracking errors, we prescribe a common set of MRMSE levels and determine, for each method, the minimum number and ratio of active radar measurements required to attain each level. A lower radiation requirement at the same prescribed MRMSE provides a more direct measure of reduced radar exposure.

During radiation, EKF-LPI, GRU-LPI, IMM-EKF-LPI, and screened IMM-GRU-LPI use EKF-CV, GRU-CV, practical IMM-EKF, and screened IMM-GRU filtering, respectively. During radar silence, their corresponding EKF-CV, GRU-CV, Wells-type IMM, and screened IMM-GRU predictors propagate the track. The neural methods use accumulated prediction variance as the radiation-restoration statistic, whereas the classical methods use the cumulative growth of the predicted position-covariance trace. Let $k_{0}$ denote the first sample of the current silent interval; the classical statistic is

(54) $$\eta_{k}=\sum_{i=k_{0}+1}^{k}\left[\mathrm{tr}(P_{i|i-1}^{p})-\mathrm{tr}(P_{i-1}^{p})\right]$$

In Equation (54), the stepwise growth of the predicted position-covariance trace is accumulated over the silent interval. The neural threshold 20,000 and classical threshold 10 were calibrated separately on validation trajectories because the two statistics have different numerical scales, and were then fixed for all tests. A maximum silent run of five samples limits open-loop error growth, while minimum on/off runs of two and three samples and a two-sample confirmation rule suppress state chattering and prevent a single noisy measurement from changing the radar state.

All four methods use the NIS in Equation (50) to terminate radiation. The threshold 10 lies near the upper tail of a two-degree-of-freedom chi-square consistency test, allowing occasional measurement fluctuations while requiring strong filter-measurement agreement before silent prediction resumes. It is fixed across all scenarios and prescribed-MRMSE levels.

All algorithms are evaluated over the same radiation-count grid, four scenarios, and five independent Monte Carlo runs at each grid point. For each method and scenario, the MRMSE sequence is ordered by radiation count and replaced by its cumulative-minimum envelope. Linear interpolation is then used to estimate the smallest active-measurement count at which each prescribed MRMSE level is attained. The common levels are 30, 40, 50, 80, 100, 150, 200, and 250 m. If a prescribed level is not attained at the largest tested count, the corresponding radiation requirement is reported as a conservative lower bound (≥) rather than extrapolated beyond the tested range.

**Figure 12:**
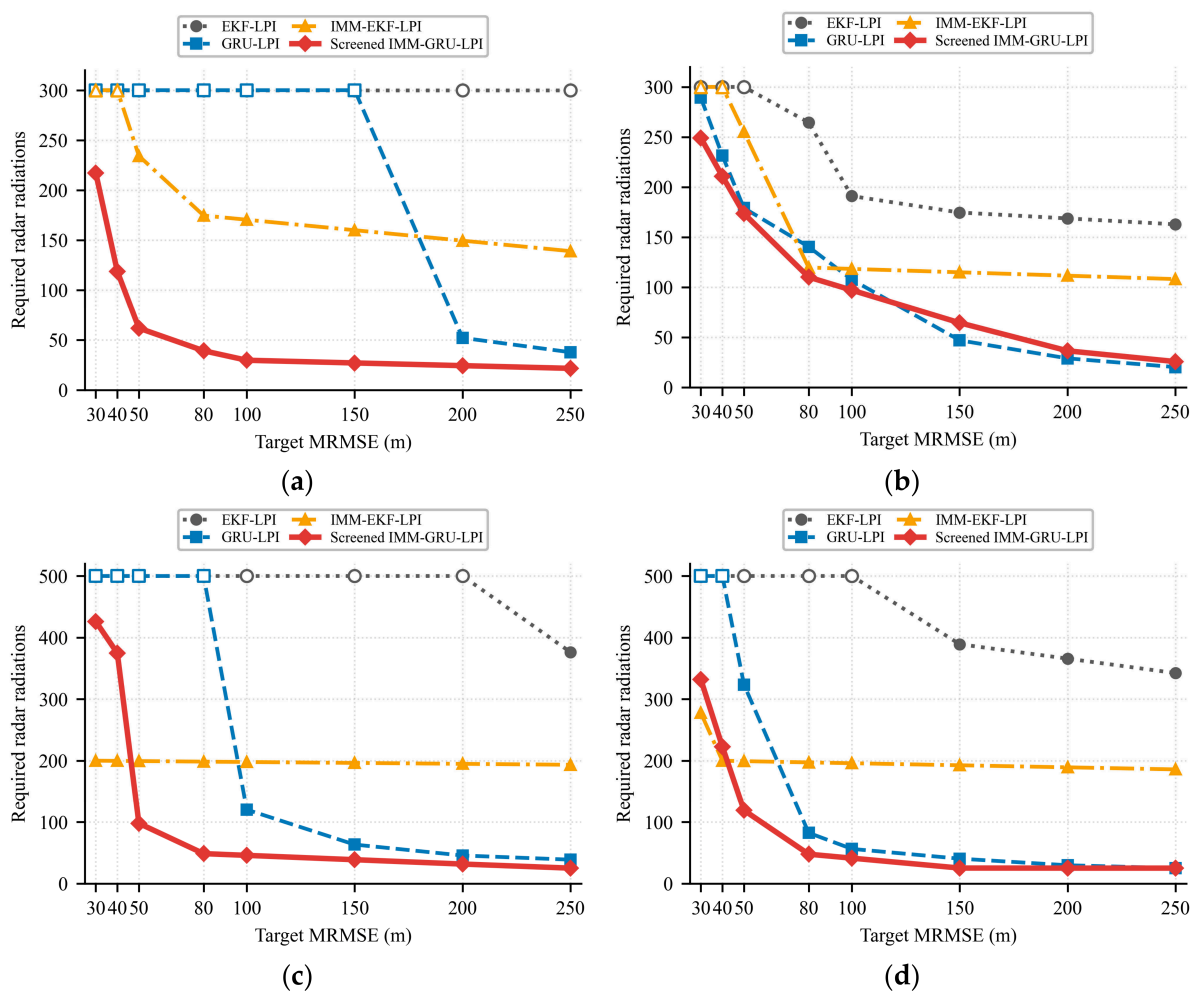
Active radar-measurement counts required to attain the prescribed MRMSE levels in Scenarios 1–4. Open markers denote MRMSE levels that are not attained within the tested radiation-count range. (**a**) Scenario 1; (**b**) Scenario 2; (**c**) Scenario 3; (**d**) Scenario 4.

Figure 12 shows that the required number of active measurements decreases monotonically as the prescribed MRMSE is relaxed. At the most stringent level of 30 m, screened IMM-GRU-LPI is the only method that satisfies the accuracy requirement in all four scenarios; the other methods contain censored points at the tested upper limit. At the remaining MRMSE levels, the proposed method generally requires substantially fewer active measurements, although GRU-LPI requires slightly fewer measurements at the three least stringent levels in Scenario 2. The scenario-wise curves therefore demonstrate the overall advantage of the proposed scheduler while also identifying the limited conditions under which a simpler single-network predictor remains competitive.

Table 9 expresses the same result as a radiation ratio, obtained by dividing the required active-measurement count by the 400 available evaluation samples. Screened IMM-GRU-LPI yields the lowest four-scenario average ratio at every prescribed MRMSE level from 40 to 250 m and is the only method with a finite four-scenario average at 30 m. Relative to the smallest competing average, its reductions at 40, 50, 80, 100, 150, 200, and 250 m are 7.3%, 49.1%, 64.4%, 63.4%, 65.6%, 24.9%, and 20.0%, respectively; comparisons involving a censored competitor are conservative. The equal-accuracy results therefore show directly that the proposed screened filtering-prediction loop achieves the prescribed tracking accuracy with a smaller fraction of active radar measurements.

## 6. Conclusions

This paper developed a screened IMM-GRU framework for maneuvering-target tracking under low-radiation operation. The filtering stage uses a fixed-lag CA-RTS reference and projects it onto the convex domain of four GRU filter outputs; the prediction stage combines model probability, learned uncertainty, inter-network disagreement, velocity continuity, and second-order-difference consistency to retain and fuse reliable predictors. The adapted IMM-EKF-CNN-LSTM baseline [28] improves upon conventional IMM-EKF but does not outperform the complete screened filter in any test scenario. The maximum-posterior IMM predictor adapted from Wells et al. [29] provides a stronger model-driven prediction reference, yet screened IMM-GRU achieves the lowest MRMSE in all six maneuver windows. The ablation study further shows that removing any screening component increases the error in every reported segment, with the largest average degradation occurring when prediction uncertainty is omitted. Under equal-accuracy LPI evaluation, screened IMM-GRU-LPI is the only method that satisfies the 30 m MRMSE requirement in all four scenarios and yields the lowest four-scenario average radiation ratio at every prescribed MRMSE level from 40 to 250 m. These conclusions are limited to independently generated horizontal-plane trajectories within the CV/CA/CT, transition, and time-varying-noise ranges examined here. A full three-dimensional implementation follows the distributed route specified in the corresponding method sections: range-azimuth-elevation measurements are converted to three-dimensional Cartesian coordinates in Section 2.1; the classifier, filter, and predictor interfaces are expanded and fine-tuned on spatial maneuvers in Section 2.2, Section 2.3 and Section 2.4; the CA-RTS state and convex projection are extended in Section 3.1.2; the prediction-consistency and mixture-variance calculations are evaluated on three-axis quantities in Section 3.2.2; and the covariance trace and three-degree-of-freedom NIS threshold are used in the scheduler in Section 4. This pathway preserves the screened IMM-GRU logic while accounting explicitly for spatial turns, climb/descent, and coupled turn-climb motion. Future work will validate this implementation using measured radar data and will further address unmodeled maneuvers, sensor bias, multi-target data association, and radiation-scheduling calibration.

## Figures and Tables

**Table 1:** Training-data parameter.

Parameter	Value or Range
Sampling interval *T*	0.1 s
Window length *L*	100
Initial range *d*	[1000, 10,000] m
Initial speed magnitude *v*	[100, 340] m/s
CA acceleration magnitude *a*	[10, 20] m/s^2^
CT turn rate ω	[−30, 30]°/s
Process-noise standard deviation $\sigma_{\omega }$	[1, 5] m/s^2^
Range measurement noise $\sigma_{d}$	[2, 10] m
Bearing measurement noise $\sigma_{\theta }$	[0.1, 0.5]°

**Table 2:** Network training parameters.

Network	Batch Size	Learning Rate	Training Epochs
CV/CA/CT filtering network	128	1.0 × 10^−3^	30
Maneuver filtering network	256	1.0 × 10^−3^	30
CV/CA/CT prediction network	128	1.0 × 10^−3^	10
Maneuver prediction network	128	1.0 × 10^−3^	30
Classification network	256	1.0 × 10^−4^	30

**Table 3:** Simulation scenario parameters.

Scene	Initial Position/m	Initial Velocity/(m/s)	Motion Model 1	Motion Model 2	Motion Model 3
1	(2000, 2000)	(250, 250)	CV 20 s	CT 20 s, ω=−20°/s	—
2	(7000, 4000)	(−180, 60)	CA 25 s, $a=(-10,6)\;m/s^{2}$	CV 15 s	—
3	(8000, −2000)	(−200, 150)	CV 20 s	CT 20 s, ω=−20°/s	CA 20 s, $a=(10,0)\;m/s^{2}$
4	(3000, 7500)	(160, −120)	CV 20 s	CT 15 s, ω=−12°/s	CA 25 s, $a=(8,10)\;m/s^{2}$

**Table 4:** Comparison of MRMSE values between GRU-CV and EKF-CV.

Algorithm	Scenario 1	Scenario 2	Scenario 3	Scenario 4
GRU-CV	174.76 m	28.75 m	93.20 m	39.72 m
EKF-CV	426.33 m	77.70 m	226.19 m	126.32 m

**Table 5:** Filtering-tracking MRMSE values (m).

Algorithm	Simulation Scenario			
	1	2	3	4
IMM-EKF	48.71	44.37	16.40	25.35
IMM-EKF–CNN-LSTM [28]	32.31	36.56	12.31	18.18
IMM-GRU	20.10	17.60	9.80	11.00
Screened IMM-GRU	16.62	14.24	7.43	8.08

**Table 6:** MRMSE values of maneuvering segments in each scenario (m). The arrow (→) here indicates a motion model transition.

Algorithm	Simulation Segment					
	Scenario 1	Scenario 2	Scenario 3		Scenario 4	
	CV → CT	CA → CV	CV → CT	CT → CA	CV → CT	CT → CA
IMM-EKF	48.00	34.40	20.39	9.94	12.26	13.29
IMM-EKF–CNN-LSTM [28]	34.28	30.29	14.95	7.72	11.42	10.19
IMM-GRU	26.40	20.18	11.82	10.05	13.38	10.84
Screened IMM-GRU	21.86	14.41	9.77	5.63	9.72	5.27

**Table 7:** Prediction MRMSE values of the comparison algorithms across maneuvering segments (m). The arrow (→) here indicates a motion model transition.

Algorithm	Simulation Segment					
	Scenario 1	Scenario 2	Scenario 3		Scenario 4	
	CV → CT	CA → CV	CV → CT	CT → CA	CV → CT	CT → CA
IMM-EKF	78.42	137.69	30.65	219.89	55.94	140.51
Wells et al. IMM predictor [29]	78.12	133.97	30.93	139.43	55.35	70.33
IMM-GRU	43.33	85.84	26.23	21.09	44.75	44.12
Screened IMM-GRU	17.60	14.26	22.39	18.20	39.56	21.94

**Table 8:** MRMSE of Screened IMM-GRU and its one-component-removed variants on the six maneuvering segments (m). The arrow (→) here indicates a motion model transition.

Algorithm	Simulation Segment					
	Scenario 1	Scenario 2	Scenario 3		Scenario 4	
	CV → CT	CA → CV	CV → CT	CT → CA	CV → CT	CT → CA
Screened IMM-GRU without model-probability gating	41.88	83.85	24.12	21.96	41.27	47.52
Screened IMM-GRU without prediction uncertainty	104.86	223.20	29.99	163.86	53.86	108.22
Screened IMM-GRU without inter-network disagreement $D_{i}$	36.66	80.74	23.37	20.70	40.81	36.41
Screened IMM-GRU without velocity-continuity $S_{i}$	43.99	80.99	24.68	22.11	41.79	46.84
Screened IMM-GRU without second-order-difference consistency $A_{i}$	44.37	83.16	24.22	21.97	41.76	51.60
Screened IMM-GRU	17.60	14.26	22.39	18.20	39.56	21.94

**Table 9:** Four-scenario average radar-radiation ratios required to attain eight prescribed MRMSE levels. The symbol ≥ denotes a conservative lower bound when at least one scenario does not attain the specified level within the tested range.

Algorithm	Prescribed MRMSE (m)							
	30 m	40 m	50 m	80 m	100 m	150 m	200 m	250 m
EKF-LPI	≥100.0%	≥100.0%	≥100.0%	≥97.8%	≥93.2%	≥85.2%	≥83.4%	≥73.8%
GRU-LPI	≥99.3%	≥95.7%	≥81.4%	≥63.9%	≥36.5%	≥28.2%	9.8%	7.6%
IMM-EKF-LPI	≥67.4%	≥62.5%	55.5%	43.1%	42.7%	41.5%	40.3%	39.2%
IMM-GRU-LPI	76.5%	58.0%	28.3%	15.3%	13.4%	9.7%	7.3%	6.1%

## Data Availability

The original contributions presented in this study are included in the article. Further inquiries can be directed to the corresponding author.

## Abbreviations

The following abbreviations are used in this manuscript:

LPI	Low Probability of Intercept
IMM	Interacting Multiple Model
EKF	Extended Kalman Filter
RNN	Recurrent Neural Network
GRU	Gated Recurrent Unit
Bi-GRU	Bidirectional Gated Recurrent Unit
CA-RTS	Continuous-Acceleration Rauch-Tung-Striebel
NIS	Normalized Innovation Squared
MRMSE	Multi-Monte-Carlo Mean Root Mean Square Error
